# Assisted reproductive technology and hypertensive disorders of pregnancy: systematic review and meta-analyses

**DOI:** 10.1186/s12884-021-03938-8

**Published:** 2021-06-28

**Authors:** Hui Ju Chih, Flavia T. S. Elias, Laura Gaudet, Maria P. Velez

**Affiliations:** 1 Department of Obstetrics and Gynecology, Kingston General Hospital, Queen’s University, Victory 4 76 Stuart St, Kingston, Ontario K7L 2V7 Canada; 2 Health Technology Assessment Program, Oswaldo Cruz Foundation, Av. Brasil, 4365 - Manguinhos, Rio de Janeiro, RJ 21040-900 Brazil; 3 Department of Public Health Sciences, Queen’s University, 62 Fifth Field Company Lane, Kingston, Ontario K7L 3N6 Canada

**Keywords:** Assisted reproductive technology, In vitro fertilization, Intracytoplasmic sperm injection, Hypertensive disorders of pregnancy, Preeclampsia, Frozen embryo transfer, Fresh embryo transfer, Oocyte donation, Meta-analysis

## Abstract

**Background:**

Hypertensive disorders of pregnancy (HDP) is one of the most common pregnancy complications and causes of maternal morbidity and mortality. Assisted reproductive technology (ART) has been associated with adverse pregnancy outcomes, including HDP. However, the impact of multiple pregnancies, oocyte donation, as well as fresh and frozen embryo transfer needs to be further studied. We conducted a systematic review and meta-analyses to evaluate the association between ART and HDP or preeclampsia relative to spontaneous conception (SC).

**Methods:**

We identified studies from EMBASE, MEDLINE, and Cochrane Library (up to April 8, 2020) and manually using structured search strategies. Cohort studies that included pregnancies after in vitro fertilization (IVF) with or without intracytoplasmic sperm fertilization (ICSI) relative to SC with HDP or preeclampsia as the outcome of interest were included. The control group was women who conceived spontaneously without ART or fertility medications. The pooled results were reported in odds ratios (OR) with 95% confidence intervals based on random effects models. Numbers needed to harm (NNH) were calculated based on absolute risk differences between exposure and control groups.

**Results:**

Eighty-five studies were included after a screening of 1879 abstracts and 283 full text articles. Compared to SC, IVF/ICSI singleton pregnancies (OR 1.70; 95% CI 1.60–1.80; I^2^ = 80%) and multiple pregnancies (OR 1.34; 95% CI 1.20–1.50; I^2^ = 76%) were both associated with higher odds of HDP. Singleton pregnancies with oocyte donation had the highest odds of HDP out of all groups analyzed (OR 4.42; 95% CI 3.00–6.51; I^2^ = 83%). Frozen embryo transfer resulted in higher odds of HDP (OR 1.74; 95% CI 1.58–1.92; I^2^ = 55%) than fresh embryo transfer (OR 1.43; 95% CI 1.33–1.53; I^2^ = 72%). The associations between IVF/ICSI pregnancies and SC were similar for preeclampsia. Most interventions had an NNH of 40 to 100, while singleton and multiple oocyte donation pregnancies had particularly low NNH for HDP (16 and 10, respectively).

**Conclusions:**

Our meta-analysis confirmed that IVF/ICSI pregnancies are at higher odds of HDP and preeclampsia than SC, irrespective of the plurality. The odds were especially high in frozen embryo transfer and oocyte donation pregnancies.

**Supplementary Information:**

The online version contains supplementary material available at 10.1186/s12884-021-03938-8.

## Background

In 2010, 48.5 million couples worldwide were estimated to be affected by infertility [1]. The use of in vitro fertilization (IVF) and other assisted reproductive technologies (ART) is expanding rapidly, accounting for more than seven million births worldwide [2]. The advancement of treatments and changes in protocols have also reshaped the landscape of fertility practice in recent years. For example, intracytoplasmic sperm injection (ICSI) is mainly indicated for male factor infertility or poor response to IVF [3]; cryopreservation has led to the rise of frozen embryo transfer (FET), which expands the scope of treatment and decreases the risk of ovarian hyperstimulation syndrome [4]; finally, oocyte donation (OD) allows women with decreased ovarian reserve or ovarian failure to achieve pregnancy [5].

While ART continues to benefit many couples around the world, it may be associated with adverse outcomes, including hypertensive disorders of pregnancy (HDP) [6–10]. HDP, including gestational hypertension and preeclampsia, occur in approximately 12–22% of all pregnancies and is associated with significant maternal and prenatal morbidity and mortality [11]. Preeclampsia is associated with a wide range of complications related to microangiopathy, vasoconstriction, and malperfusion. Women with a history of preeclampsia also continue to be at a high risk for cardiovascular disease, chronic kidney disease, and cardiovascular mortality even after pregnancy [12]. The pregnancy and postpartum complications as well as high mortality rates highlight the importance of prevention and early detection of HDP.

Although previous meta-analyses have shown that ART is associated with an increased risk of preeclampsia, the underlying mechanism is not well understood [13, 14]. Many included studies were based on singleton pregnancies or mixed cohorts, while studies specifically comparing the risk of HDP in ART and spontaneous multiple pregnancies often yielded inconsistent results [9, 15]. The types of ART and treatment protocols also appear to play a role in differences in maternal and perinatal outcomes [16]. For example, recent meta-analysis by Rogue et al. showed that FET is associated with a higher rate of low birth weight and preeclampsia when compared to fresh ET [17]. Currently, it remains unclear whether the differences in pregnancy outcomes, including HDP, were due to maternal factors, the procedure itself, or both. With the increasing number of literature over the past decade and changes in protocols, there is a need for an updated and comprehensive review of IVF/ICSI pregnancies in consideration of patient and treatment factors.

Our systematic review and meta-analysis aim to understand whether IVF/ICSI pregnancies are associated with increased odds of HDP and preeclampsia in comparison to spontaneous conception (SC); furthermore, we aim to understand whether the odds differ depending on types of procedure. Together, this review may inform clinical recommendations for women planning to achieve pregnancy through IVF/ICSI.

## Methods

### Search strategy

The study follows the Preferred Reporting Items for Systematic Reviews and Meta-Analysis (PRISMA) checklist (Supplementary information, Additional file 1) and the protocol was registered and available on Open Science Framework (DOI: https://osf.io/562jr/). A search strategy was developed under the support of a research librarian to identify studies evaluating the incidence of HDP and/or preeclampsia in IVF or ICSI pregnancies compared to SC (Additional file 2). MeSH terms and selection criteria were based on the Patient, Intervention, Comparison and Outcome statement. Cohort studies published up to April 8, 2020 were retrieved from Medline, Embase, and Cochrane Central Register of controlled Trials using the OVID platform. A manual search of previously published systematic reviews and meta-analysis was also conducted to identify other eligible studies.

### Selection of studies

Both abstract and full text screening were performed by two reviewers (HC, FTSE). In the first screening, articles were selected based on titles and abstracts. The second screening involved full-text reviews, where studies were evaluated based on a set of eligibility criteria. Any conflict was resolved by consensus or the involvement of a third team member (MPV).

Studies that compared pregnancies after IVF or ICSI and SC with HDP or preeclampsia as the outcome of interest were included. The control group consisted of women who conceived spontaneously without the use of ART or fertility medications. The exposure group consisted of singleton or multiple IVF/ICSI pregnancies. Non-randomized studies in the form of prospective and retrospective cohort studies were of interest; other study designs such as review articles, randomized control trials, case-control studies, conference abstracts, and case reports were excluded. Studies were excluded if they were not in English, French, Portuguese, or Chinese, included patients undergoing ART or fertility treatments other than IVF/ICSI, did not specify the type of ART used, or did not clearly separate patients into singleton or multiple pregnancies. Studies that included a subgroup of women (e.g. advanced maternal age, obesity) were not included in the general singleton and multiple gestation analyses as they were not representative of the general population. However, they were included in sub-analyses for type of embryo transfer (fresh embryo transfer (fresh ET) or FET) and OD. For studies with overlapping cohorts, where the same database was used for analyses, only the most recent study was included in the meta-analysis. A complete list of excluded studies after full text screening with their respective reasons of exclusion may be found in Additional file 3.

Outcomes of interest included HDP and preeclampsia. Hypertensive disorders of pregnancy describe any hypertensive effects that is observed during pregnancy, including pre-existing hypertension, gestational hypertension and preeclampsia. Preeclampsia was defined as hypertension that develops for the first time after 20 weeks of gestation with one or more of the following: proteinuria, adverse conditions, or severe complications [18].

### Data extraction and quality assessment

Data was extracted manually and entered into an Excel spreadsheet by a reviewer (HC). The following characteristics of each study were collected: authorship, year of publication, country, study design, search database, time period of the cohort, matching factors, statistical analysis, outcome of interest, definition of outcome, mean maternal age, mean BMI, number of patients with chronic hypertension, type of ART, type of infertility, source of oocyte, method of embryo transfer, sample size, and crude data. If needed, percentages of HDP and preeclampsia were converted to crude data based on the sample size. Study quality was assessed using the Newcastle-Ottawa Scale for Cohort Studies [19]. Each study was scored out of nine based on eight items across three domains: the selection of study groups (4 items), comparability of groups (1 item), and ascertainment of exposure or outcome of interest in cohort studies, respectively (3 items). It was then determined to have either high quality (8 or 9), moderate quality (6 or 7), or low quality (less than 5) based on the total NOS score. A second reviewer (FTSE) reviewed all data extraction and quality assessment performed.

### Statistical analysis

The meta-analyses were performed using Review Manager (RevMan) version 5.4. Cohort studies were included in the general meta-analyses by plurality. Studies that explicitly excluded ICSI pregnancies and those that included ICSI pregnancies only were included in the IVF and ICSI sub-analyses, respectively. In addition, separate analyses on fresh ET, FET, and OD were also conducted. Results were reported as odds ratios with corresponding 95% confidence intervals based on random effects models, which assumed heterogeneity of the data. The Mantel-Haenszel method was used to calculate overall odds ratios. Statistical significance was determined by a *P* value of equal or less than 0.05. Numbers needed to harm (NNH), which represented the number of patients needed to undergo IVF/ICSI for one patient to receive harm, were calculated based on the absolute risk differences between exposure and control groups [20]. Sensitivity analysis was carried out by removing one study at a time to assess the effect of the study on the results. If the measure of association without the chosen study fell outside of the confidence interval, the study was said to have a significant influence [21]. I-squared (I^2^) test was used to evaluate heterogeneity, with an I^2^ value of greater than 50% being considered as high heterogeneity [22]. Risk of publication bias was evaluated using funnel plots if the meta-analysis included 10 or more studies [23].

## Results

### Study selection

**Fig. 1:**
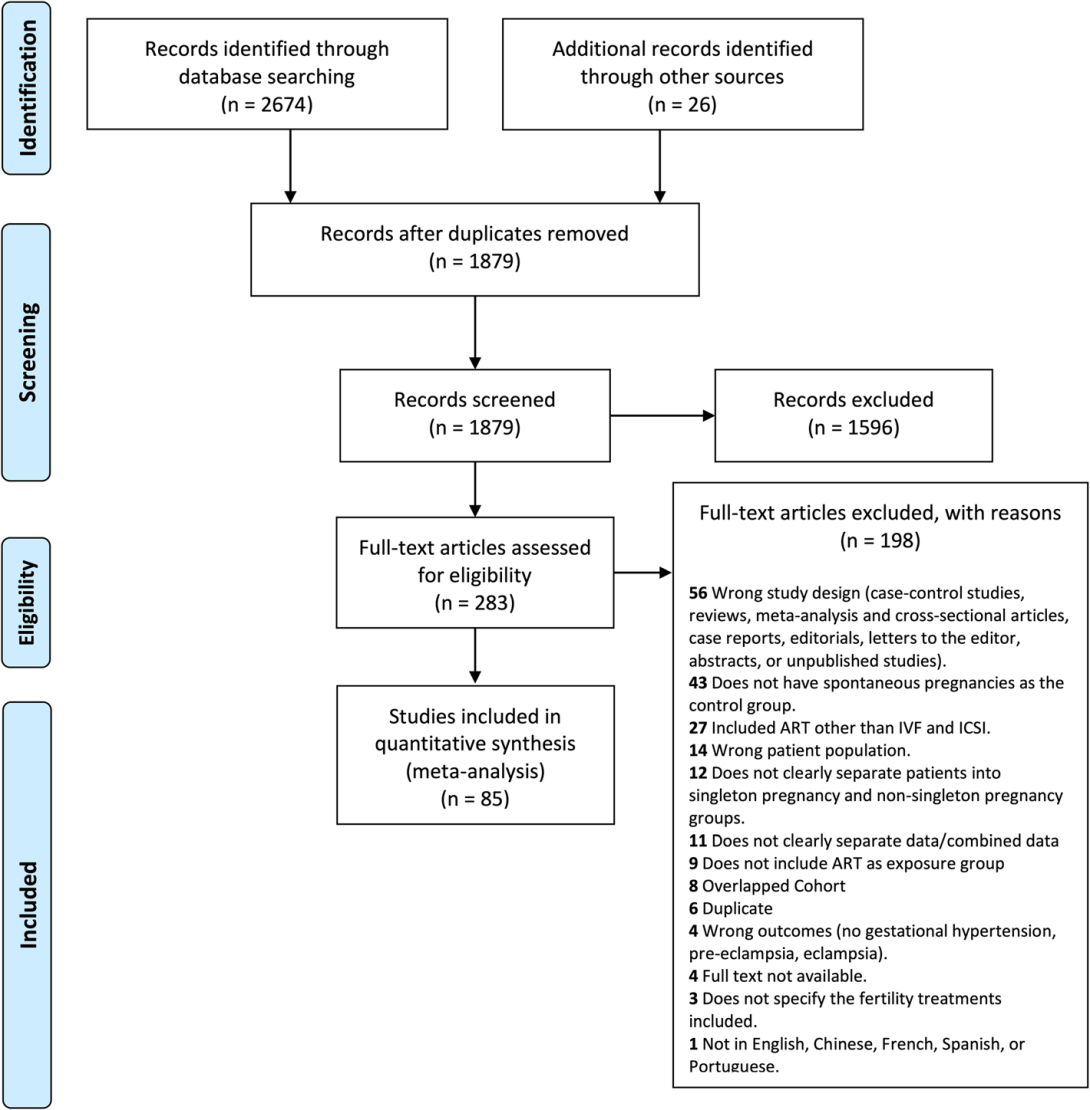
PRISMA Flowchart. Flow diagram for study identification and inclusion according to the Preferred Reporting Items for Systematic Reviews and Meta-Analyses (PRISMA) guidelines

Our search strategy identified 2674 studies and 1879 citations were eligible for abstract and title screening after removing duplicates. Finally, 85 studies met the inclusion criteria and were included in the meta-analysis (Fig. 1). Eight studies had overlapping cohorts with more recently published studies and thus were excluded (Additional file 3).

### Characteristics of included studies

The characteristics of all included studies, which involved 405,920 IVF/ICSI pregnancies and 8,122,210 SC, may be found in Table 1. The sizes of the exposure (IVF/ICSI) and control (SC) groups ranged from 19 to 83,582 pregnancies and from 21 to 1,382,311 pregnancies respectively. Out of the 85 studies included, 21 were population-based cohort studies conducted in Canada [39, 40, 88], Denmark [64], Finland [55, 78], Israel [73, 85], China [105], Japan [69], Netherlands [31], Norway [94], Slovenia [52], Sweden [43, 44, 71, 83], and the United States [62, 63]. Two studies were conducted across multiple European countries [75, 101]. Fifty-one studies looked at the incidences of preeclampsia or HDP in IVF/ICSI singleton pregnancies in comparison to SC, while 41 studies investigated the outcomes of multiple pregnancies in particular (Table 1). Based on their respective NOS scores, 15 studies had a high methodological quality, 61 studies had a moderate quality, and 9 had a low quality (Table 1, Additional file 4). Thirty-three studies were matched cohort studies using varying factors such as maternal age, birth year, parity, socioeconomic status, location (Table 1). Eight studies used chronic hypertension to adjust the comparability between exposure and control groups [65, 70, 75, 81, 88, 97, 100, 108]. One study calculated propensity scores that account for 27 maternal and paternal variables [100].

**Table 1:** Characteristics of 78 included cohort studies. S = Singleton pregnancy, M = Multiple pregnancy

First author, Publication year, Country	Type of cohort	Years of the cohort	Matching factors	Comparison groups	Pregnancies conceived by IVF/ICSI (n)	Spontaneous pregnancies (n)	NOS Score
Agarwal, 2005, Singapore [24]	Hospital-based Retrospective	1998–1999	Maternal age, sex, date of delivery, race, plurality and parity	IVF/ICSI, ICSI alone	(S) 41 (M) 35	(S) 147 (M)114	7
Ai, 2005, China [25]	Hospital-based Retrospective	1998–2004	No	IVF/ICSI	(M) 47	(M) 98	6
Apantaku, 2008, UK [26]	Hospital-based Retrospective	1999–2004	Maternal age, parity	IVF/ICSI	(S) 88	(S) 88	8
Aydin, 2016, Turkey [27]	Hospital-based Retrospective	2007–2010	Maternal age	IVF/ICSI	(M)137	(M) 133	8
Barda, 2017, Israel [28]	Hospital-based Retrospective	2009–2015	No	IVF/ICSI	(M) 449	(M) 259	6
Barua, 2016, Australia [29]	Hospital-based Retrospective	2007–2010	No	IVF/ICSI, ICSI alone	(S) 470	(S) 48654	6
Beltran Anzola, 2019, France [30]	Hospital-based Retrospective	1995–2015	Maternal age, exact year of birth, parity, sex	IVF/ICSI, Fresh embryo transfer, Frozen embryo transfer	(S) 2327	(S) 6981	7
Bensdorp, 2016, Netherlands [31] (a)	Population-based Retrospective	2000–2012	Zygosity, parity, socioeconomic status, conception method	IVF/ICSI, ICSI alone	(M) 2437	(M) 3276	7
Beyer, 2016, Germany [32]	Hospital-based Retrospective	N/A (13-year period)	No	IVF/ICSI, Fresh embryo transfer, Frozen embryo transfer	(S) 467	(S) 6417	6
Carbone, 2011, UK [33]	Hospital-based Prospective	2006–2009	No	IVF/ICSI	(S) 426	(S) 26538	7
Caserta, 2008, Italy [34]	Hospital-based Prospective	2004–2006	Parity, age, height, weight, ethnic origin, smoking, history of infertility	IVF/ICSI, ICSI alone	(S) 364	(S) 304	3
Caserta, 2014, Italy [35]	Hospital-based Retrospective	2007–2011	No	IVF/ICSI	(M) 138	(M) 207	7
Choi, 2006, Korea [36]	Hospital-based Retrospective	1994–2003	No	IVF/ICSI	(M) 190	(M) 347	6
Daniel, 2000, Israel [37]	Hospital-based Retrospective	1996–1997	No	IVF/ICSI	(M) 104	(M) 121	7
Dayan, 2015, Canada [38]	Hospital-based Retrospective	2001–2008	No	IVF/ICSI	(S) 326	(S) 9175	7
Dayan, 2016, Canada [39]	Population-based Retrospective	2006–2012	No	IVF/ICSI	(S) 5371	(S) 795997	7
Dayan, 2018, Canada [40]	Population-based Retrospective	2013–2014	No	IVF/ICSI	(S) 1596	(S) 112813	6
Deltombe-Bodart, 2017, France [41]	Hospital-based Retrospective	1997–2014	No	IVF/ICSI, ICSI alone	(M) 360	(M) 986	6
Dior, 2018, Israel [42]	Hospital-based Retrospective	1995–2012	No	Oocyte Donation	(S) 135	(S) 270	7
Elenis, 2015, Sweden [43]	Population-based Retrospective	2005–2008	Age	IVF/ICSI, Oocyte Donation	(S) 139	(M) 150	7
Ernstad, 2019, Sweden [44]	Population-based Retrospective	2005–2015	No	IVF/ICSI, Fresh embryo transfer	(S) 34091	(S) 1127566	6
Fan, 2013, China [45]	Hospital-based Retrospective	2010–2013	No	IVF/ICSI, ICSI alone	(M) 162	(M) 213	7
Farhi, 2013, Israel [46]	Hospital-based Prospective	2006–2008	No	IVF/ICSI, ICSI alone	(S) 509	(S) 587	5
Geipel, 2001, Germany [47]	Hospital-based Retrospective	1995–1999	Maternal age, parity, plurality	IVF/ICSI, ICSI alone	(S) 114 (M) 32	(S) 114 (M) 32	8
Gocmen, 2015, Turkey [48]	Hospital-based Retrospective	2011–2014	No	IVF/ICSI	(M) 19	(M) 65	8
Gojnic, 2005, Serbia [49]	N/A	N/A	Age, education, parity	IVF/ICSI	(M) 120	(M) 120	2
Hessami, 2020, Iran [50]	Hospital-based Retrospective	2013–2018	No	IVF/ICSI	(M) 202	(M) 449	6
Howe, 1990, US [51]	Hospital-based Retrospective	N/A (first 100 clinical pregnancies conceived in the IVF program)	Age, race, parity, pre-existing medical problem, DES exposure, insurance status	IVF/ICSI	(S)54	(S)54	7
Jancar, 2018, Slovenia [52]	Population-based Retrospective	2002–2015	No	IVF/ICSI	(S) 5837	(S) 261881	7
Jeve, 2016, UK [53]	Hospital-based Retrospective	2007–2014	Age	IVF/ICSI, Oocyte donation	(S) 90	(S) 45	8
Katalinic, 2004, Germany [54]	Hospital-based Prospective	1998–2000; 1993–2001 (control)	No	IVF/ICSI, ICSI alone, Fresh embryo transfer	(S) 2055 (M) 632	(S) 7861 (M) 77	6
Koivurova, 2002, Finland [55]	Population-based Retrospective	1990–1995	Sex of the child, birth year, area of residence, parity, maternal age, social class (defined by occupation)	IVF/ICSI, Fresh embryo transfer	(S) 153 (M) 62	(S) 580 (M) 82	7
Korosec, 2016, Slovenia [56]	Hospital-based Retrospective	2004–2011	Age, parity, hospital	IVF/ICSI, Fresh embryo transfer, Frozen embryo transfer	(S)1127	(S) 3381	7
Kouhkan, 2018, Iran [57]	Hospital-based Prospective	2014–2017	No	IVF/ICSI	(S) 260	(S) 314	7
Kuivasaari- Pirinen, 2012, Finland [58]	Hospital-based Retrospective	1996–2007	No	IVF/ICSI	(S) 255	(S) 26870	7
Lee, 2015, US [59]	Hospital-based Retrospective	2007–2009	No	IVF/ICSI	(S) 108	(S) 2284	7
Lei, 2019, China [60]	Hospital-based Retrospective	2013–2015	No	IVF/ICSI	(S) 1453 (M) 803	(S) 6667 (M) 101	6
Li, 2015, China [61]	Hospital-based Retrospective	2009–2011	No	IVF/ICSI	(M) 108	(M) 144	6
Luke, 2019, US [62]	Population-based Retrospective	2004–2013 (depending on states)	No	IVF/ICSI, Oocyte donation	(M) 58,920	(M) 34,033	7
Luke, 2020, US [63]	Population-based Retrospective	2004–2013 (depending on states)	No	IVF/ICSI, Fresh embryo transfer, Frozen embryo transfer, Oocyte donation	(S) 83582	(S) 1382311	6
Malchau, 2013, Denmark [64]	Population-based Retrospective	1995–2010	Date and year of birth	IVF/ICSI, ICSI alone, Oocyte donation	(S) 15741 (M) 8564	(S) 31010 (M) 25,012	7
Martinez-Varea, 2015, Spain [65]	Hospital-based Prospective	N/A	No	IVF/ICSI, Oocyte donation	(S) 50	(S) 25	6
Meyer, 2020, Israel [66]	Hospital-based Retrospective	2011–2018	Age	Oocyte donation	(S) 159	(S) 73	7
Mohammed, 2012, Qatar [67]	Hospital-based Retrospective	2002–2011	No	IVF/ICSI	(M) 145	(M) 175	7
Moini, 2012, Iran [68]	Hospital-based Prospective	2008–2010	No	IVF/ICSI, ICSI alone	(M) 230	(M) 170	6
Nagata, 2019, Japan [69]	Population-based Retrospective	2011–2014	No	IVF/ICSI, ICSI alone	(S) 2993 (M) 129	(S) 88873 (M) 625	7
Nassar, 2003, Lebanon [70]	Hospital-based Retrospective	1995–2000	Age, parity	IVF/ICSI	(M) 56	(M) 112	9
Nejdet, 2016, Sweden [71]	Population-based Retrospective	2003–2012	No	IVF/ICSI, Fresh embryo transfer, Frozen embryo transfer, Oocyte donation	(S) 27084	(S) 999804	7
Ochsenkuehn, 2003, Germany [72]	Hospital-based Retrospective	1991–1996	Maternal age, gestational age, parity	IVF/ICSI	(S) 163 (M) 65	(S) 322 (M) 78	8
Okby, 2018, Israel [73]	Population-based Retrospective	1988–2010	No	IVF/ICSI	(M) 465	(M) 3053	8
Olivennes, 1993, France [74]	Hospital-based Retrospective	1987–1989	No	IVF/ICSI	(S) 162	(S) 5096	6
Opdahl, 2015, Sweden, Denmark, Norway [75]	Population-based Retrospective	1988–2007	Parity, birth year	IVF/ICSI	(S) 47088 (M) 10,918	(S) 268599 (M) 46,674	9
Poikkeus, 2007, Finland [76]	Hospital-based Retrospective	1997–2003	Year, place of residence	IVF/ICSI, Fresh embryo transfer	(S) 499	(S) 15037	7
Qin, 2017, China [77]	Hospital-based Prospective	2013–2016	No	IVF/ICSI	(S) 1260	(S) 2480	6
Raisanen, 2013, Finland [78]	Population-based Retrospective	2006–2010	No	IVF/ICSI	(S) 5647	(S) 285357	7
Reismullerova, 2015, Slovakia [79]	Hospital-based Retrospective	N/A	No	IVF/ICSI	(S) 526	(S) 15874	7
Reubinoff, 1997, Israel [80]	Hospital-based Retrospective	1983–1993	Maternal ethnic origin, age, parity, location and date of delivery	IVF/ICSI	(S) 260	(S) 260	7
Rizzo, 2016, Italy [81]	Hospital-based Prospective	2007–2014	Maternal age	IVF/ICSI, Fresh embryo transfer, Frozen embryo transfer	(S) 266	(S) 266	9
Rizzo, 2016, Italy [2] [82]	Hospital-based Prospective	2007–2014	Maternal age	IVF/ICSI, Oocyte donation	(S) 109	(S) 498	9
Sazonova, 2012, Sweden [83]	Population-based Retrospective	2002–2006	No	IVF/ICSI, Fresh embryo transfer, Frozen embryo transfer	(S) 11292	(S) 571914	7
Shi, 2018, China [84]	Hospital-based Retrospective	2013–2016	No	IVF/ICSI	(M) 850	(M) 250	5
Shiloh, 2019, Israel [85]	Population-based Retrospective	1991–2014	No	IVF/ICSI	(S) 2603	(S) 237863	6
Silberstein, 2014, Israel [86]	Hospital-based Retrospective	1988–2006	No	IVF/ICSI	(S) 1294	(S) 171513	6
Stojnic, 2013, Serbia [87]	Hospital-based Retrospective	2006–2010	Maternal age, parity, education, time and place of delivery, BMI	IVF/ICSI, Fresh embryo transfer	(S) 634	(S)634	7
Sun, 2009, Canada [88]	Population-based Retrospective	2004–2007	Maternal age, parity	IVF/ICSI	(S) 870	(S) 3433	9
Sun, 2016, China [89]	Hospital-based Retrospective	2010–2014	No	IVF/ICSI	(M) 411	(M) 742	7
Suzuki, 2010, Japan [90]	Hospital-based Retrospective	2000–2007	No	IVF/ICSI	(M) 64	(M) 76	6
Szymusik, 2012, Poland [91]	Hospital-based Retrospective	2005–2009	No	IVF/ICSI, Fresh embryo transfer	(M) 43	(M)83	4
Szymusik, 2018, Poland [92]	Hospital-based Prospective	2013–2016	No	IVF/ICSI	(S) 183	(S) 368	4
Tan, 1992, UK [93]	Hospital-based Retrospective	1978–1987	Maternal age	IVF/ICSI	(S) 494 (M) 125	(S) 978 (M) 21	6
Tandberg, 2015, Norway [94]	Population-based Retrospective	1988–2009	Parity	IVF/ICSI	(S) 12440	(S) 1097084	8
Tomic, 2011, Croatia [95]	Hospital-based Retrospective	2006–2009	Ethnicity, age, gravidity, smoking habits, BMI, weight gain in pregnancy, site and time of delivery	IVF/ICSI, Fresh embryo transfer	(S) 283	(S) 283	7
Valenzuela-Alcaraz, 2013, Spain [96]	Hospital-based Prospective	N/A	Age	IVF/ICSI	(S) 100	(S) 100	6
Valenzuela-Alcaraz, 2018, Spain [97]	Hospital-based Prospective	2014–2016	No	IVF/ICSI	(M) 50	(M) 50	7
Vasario, 2012, Italy [98]	Hospital-based Prospective	2004–2008	No	IVF/ICSI	(M) 84	(M) 139	6
Von Versen-Hoynck, 2019, US [99]	Hospital-based Prospective	2011–2017	No	IVF/ICSI, Fresh embryo transfer, Frozen embryo transfer	(S) 367	(S)143	5
Watanabe, 2014, Japan [100]	Hospital-based Retrospective	2009–2011	Closest propensity score (accounting for 27 maternal and paternal variables)	IVF/ICSI	(S) 474	(S) 474	9
Wennberg, 2016, Sweden, Denmark, Finland, Norway [101]	Population-based Retrospective	1982–2007	Parity, year and month of birth	IVF/ICSI, Fresh embryo transfer	(S) 39919	(S) 260166	7
Wu, 2010, China [102]	Hospital-based Retrospective	2006–2008	No	IVF/ICSI	(M) 204	(M) 255	4
Xu, 2005, China [103]	Hospital-based Retrospective	2001–2003	No	IVF/ICSI	(M) 41	(M) 44	4
Yang, 2011, Korea [104]	Hospital-based Retrospective	1995–2008	No	IVF/ICSI	(M) 67	(M) 143	7
Yang, 2014, China [105]	Population-based Retrospective	2011	No	IVF/ICSI	(S) 825 (M) 314	(S) 109971 (M) 1473	6
Zadori, 2003, Hungary [106]	Hospital-based Retrospective	1995–2002	Maternal age, parity, gravidity, previous obstetrics outcomes	IVF/ICSI	(S) 185 (M) 36	(S) 185 (M) 36	6
Zhang, 2015, China [107]	Hospital-based Retrospective	2010–2014	No	IVF/ICSI	(M) 53	(M) 128	6
Zhu, 2016, China [108]	Hospital-based Retrospective	2006–2014	Maternal age, birth year	IVF/ICSI	(S) 1659 (M) 982	(S) 5193 (M) 89	9

### Specific outcomes

#### IVF/ICSI singleton pregnancies

**Fig. 2:**
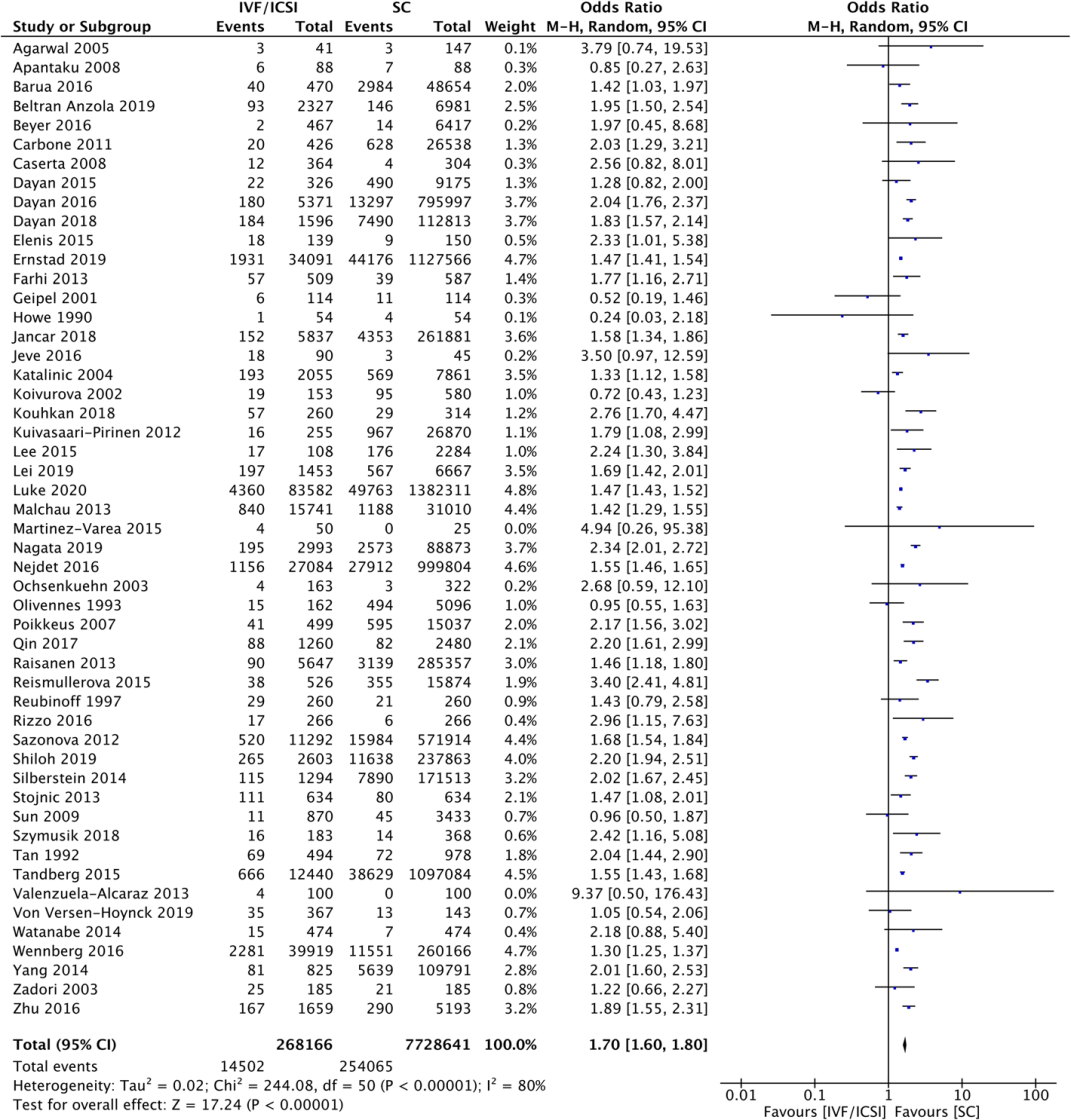
IVF/ICSI Singleton Pregnancies Meta-analysis. Forest plot comparing hypertensive disorders of pregnancy in IVF/ICSI singleton pregnancies in comparison to spontaneous pregnancies

**Fig. 3:**
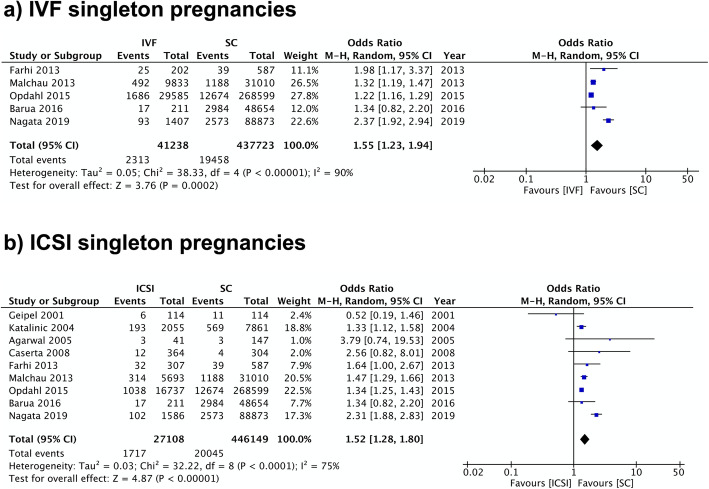
IVF and ICSI Singleton Pregnancies Meta-analysis. Forest plot comparing hypertensive disorders of pregnancy in **a**) IVF singleton pregnancies and **b**) ICSI singleton pregnancies in comparison to spontaneous pregnancies

Fifty-one studies including 268,166 pregnancies in the IVF/ICSI group and 7.7 million pregnancies in the SC group were included in the analysis of HDP. The overall odds ratio (OR) was 1.70 (95% CI 1.60–1.80) with high heterogeneity (I^2^ = 80%) (Fig. 2). Almost all studies were of high or moderate quality according to their NOS scores; four were categorized as low quality. A separate analysis of 5 studies that included only IVF pregnancies yielded consistent findings with an OR of 1.55 (95% CI 1.23–1.94; I^2^ = 90%) (Fig. 3).

Of those studies above, 28 studies specifically reported the incidence of preeclampsia, resulting in a sample size of 118,787 pregnancies in the IVF/ICSI group and 4.5 million pregnancies in the SC group. Cumulative incidences found that the IVF/ICSI group had significantly higher odds of preeclampsia than the SC group with an OR of 1.59 (95% CI 1.46–1.74) with high heterogeneity (I^2^ = 70%) (Additional file 5). Twenty-seven studies were classified as high or moderate quality, and one study received an NOS score of low quality. Half of the included studies matched the control group with IVF/ICSI group by maternal factors such as age or parity. Only one study studying preeclampsia explicitly excluded ICSI pregnancies and therefore a sub-analysis for IVF could not be conducted.

The sub-analysis of nine studies that included ICSI pregnancies only found that this type of procedure had a higher rate of HDP in comparison to SC. The resulting OR was 1.52 (95% CI 1.28–1.80; I^2^ = 75%) (Fig. 3). In the case of preeclampsia, only two studies were eligible with no significant difference between the two groups (OR 0.98, 95% CI 0.38–2.51; I^2^ = 72%) (Additional file 5). Both pooled analyses showed high heterogeneity.

#### IVF/ICSI multiple pregnancies

**Fig. 4:**
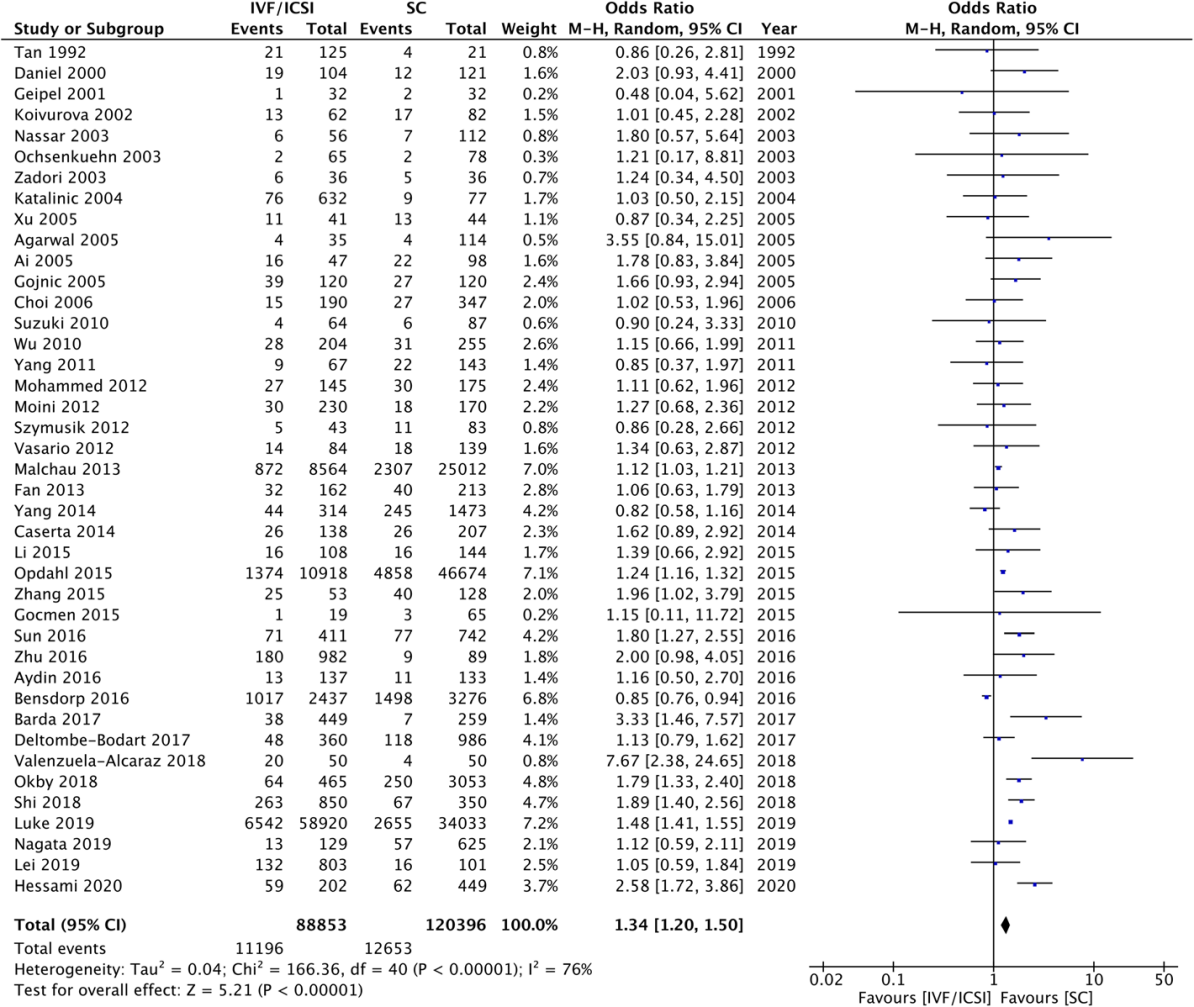
IVF/ICSI Multiple Pregnancies Meta-analysis. Forest plot comparing hypertensive disorders of pregnancy in IVF/ICSI multiple pregnancies in comparison to spontaneous pregnancies

Forty-one studies assessed HDP in multiple pregnancies. Higher odds were observed in the IVF/ICSI group than the SC group, with an OR of 1.34 (95% CI 1.20–1.50) with high heterogeneity (I^2^ = 76%) (Fig. 4). The number of studies that were rated as high, moderate, and low quality by NOS scores were 8, 28, and 5 respectively.

Seventeen studies assessed preeclampsia as the outcome of interest. The odds of preeclampsia were higher in pregnancies resulting from IVF/ICSI than SC (OR 1.24, 95% CI 1.08–1.43) with low heterogeneity (I^2^ = 32%) (Additional file 5). All studies either had moderate or high quality.

**Fig. 5:**
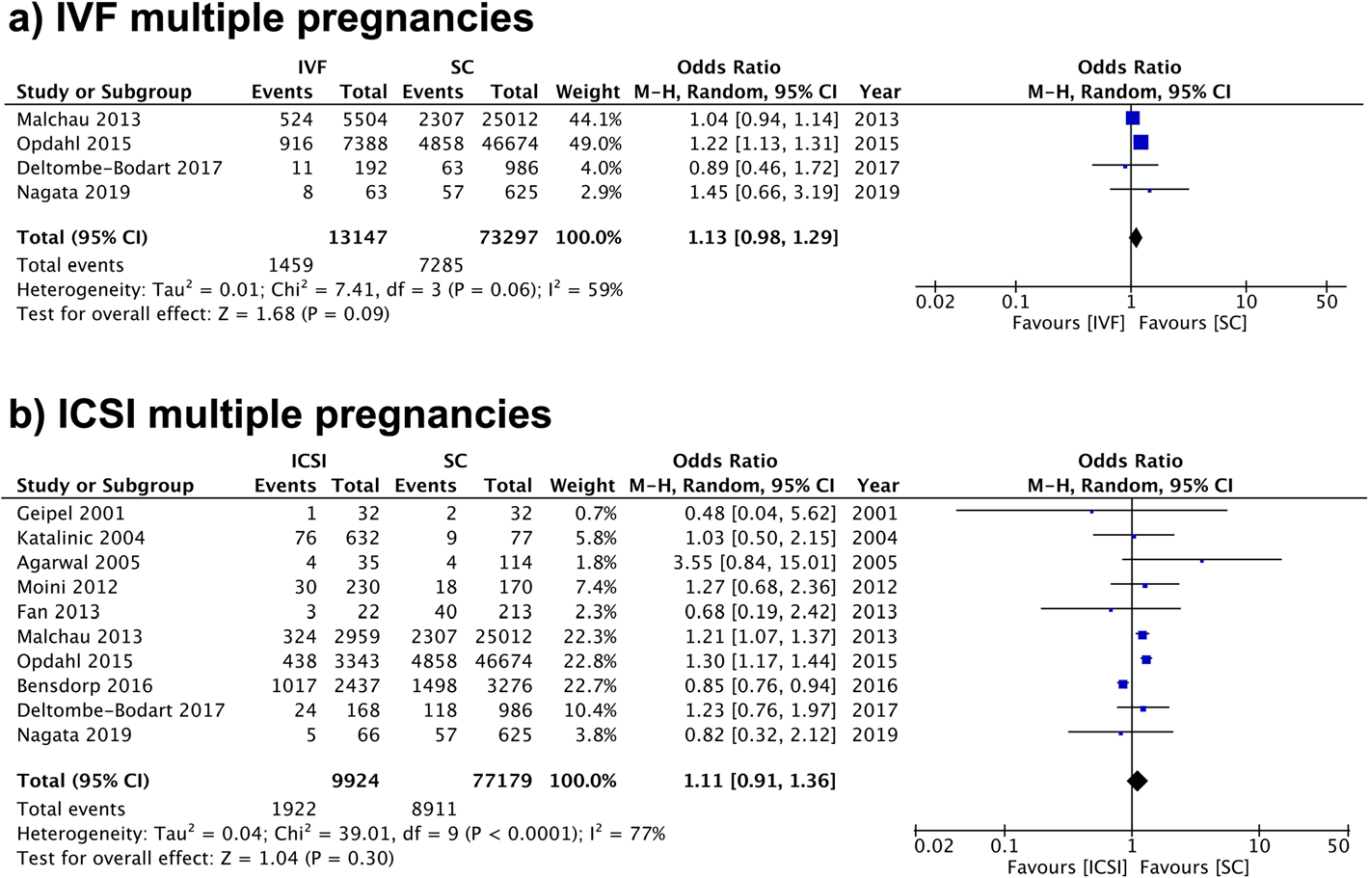
IVF and ICSI Multiple Pregnancies Meta-analysis Forest plot comparing hypertensive disorders of pregnancy in **a**) IVF multiple pregnancies and b) ICSI multiple pregnancies in comparison to spontaneous pregnancies

For IVF pregnancies, four and two studies looked at HDP and preeclampsia respectively. Both showed slightly increased odds in the ART groups in comparison to SC; however, the differences were insignificant (HDP: OR 1.13, 95% CI 0.98–1.29; I^2^ = 59%. Preeclampsia: OR 1.04, 95% CI 0.93–1.16; I^2^ = 0%) (Fig. 5, Additional file 5).

For ICSI multiple pregnancies, 10 studies were eligible for the analysis of HDP. Overall, no increase in the risk of HDP was observed in the exposure group (OR 1.11, 95% CI 0.91–1.36; I^2^ = 77%) (Fig. 3). Five studies reported data on preeclampsia in multiple pregnancies after ICSI. The odds of preeclampsia were slightly higher in pregnancies resulting from ICSI than SC (OR 1.11, 95% CI 1.00–1.24) in the pooled analysis with no heterogeneity (I^2^ = 0%) (Additional file 5).

#### Fresh and frozen embryo transfer

**Fig. 6:**
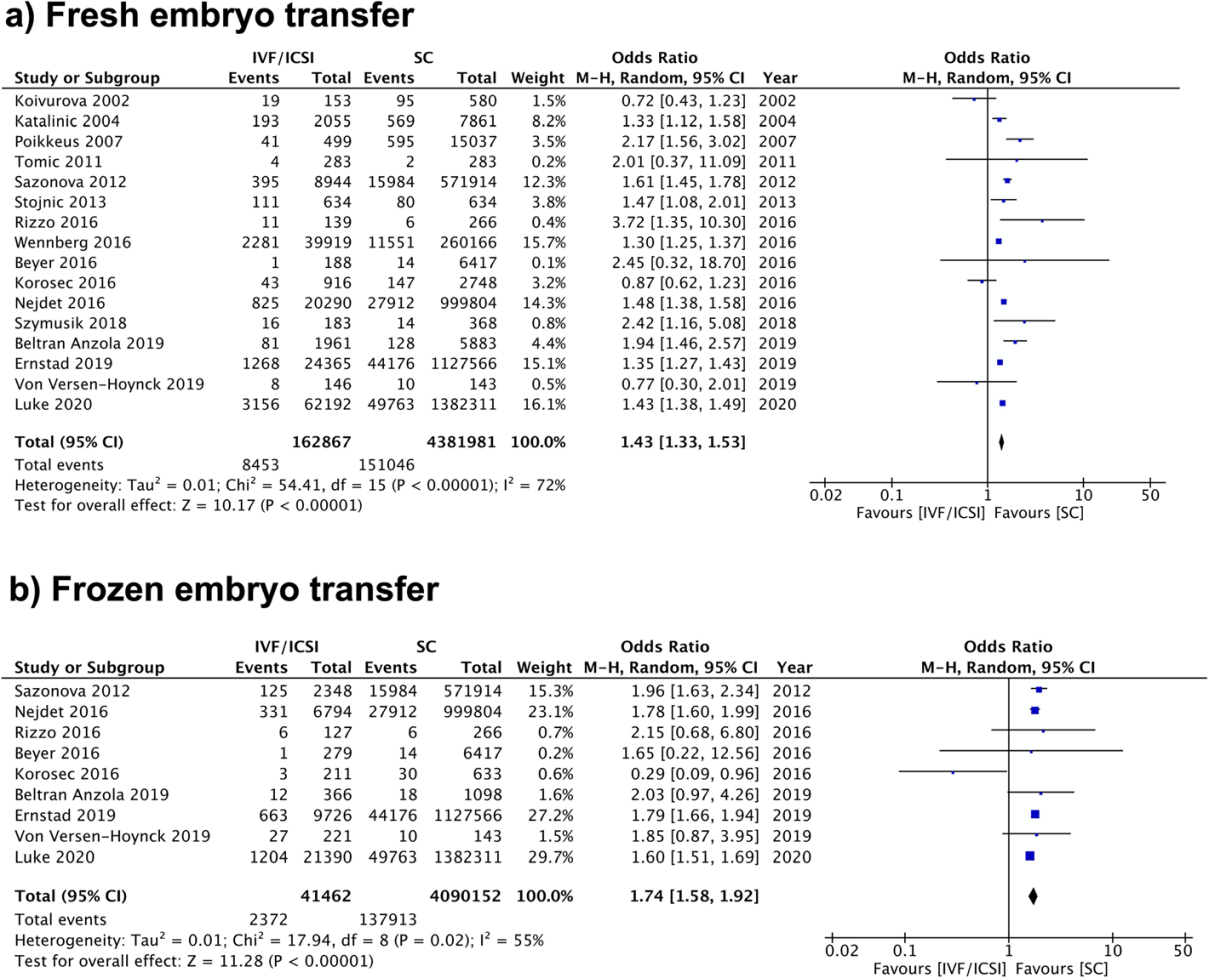
Fresh and Frozen Embryo Transfer Meta-analysis. Forest plot comparing hypertensive disorders of pregnancy in singleton pregnancies resulting from **a**) fresh embryo transfer or **b**) frozen embryo transfer in comparison to spontaneous pregnancies

Sixteen studies reported on the relationship between fresh ET and HDP. The pooled result showed that, when compared to SC, fresh ET is associated with increased odds of HDP with an OR of 1.43 (95% CI 1.33–1.53; I^2^ = 72%) (Fig. 6). A similar finding was also found in the pooled result of eight studies using preeclampsia as the outcome of interest (OR 1.48, 95% CI 1.37–1.60) with low heterogeneity (I^2^ = 39%) (Additional file 5).

FET was also associated with higher odds of HDP and preeclampsia compared to SC. Nine studies were included resulting in a pooled OR of 1.74 (95% CI 1.58–1.92; I^2^ = 55%) for HDP (Fig. 6). Comparably, in five studies that studied preeclampsia, the OR was 1.82 (95% CI 1.71–1.95) with no heterogeneity between included studies (I^2^ = 0%) (Additional file 5).

#### Oocyte donation

**Fig. 7:**
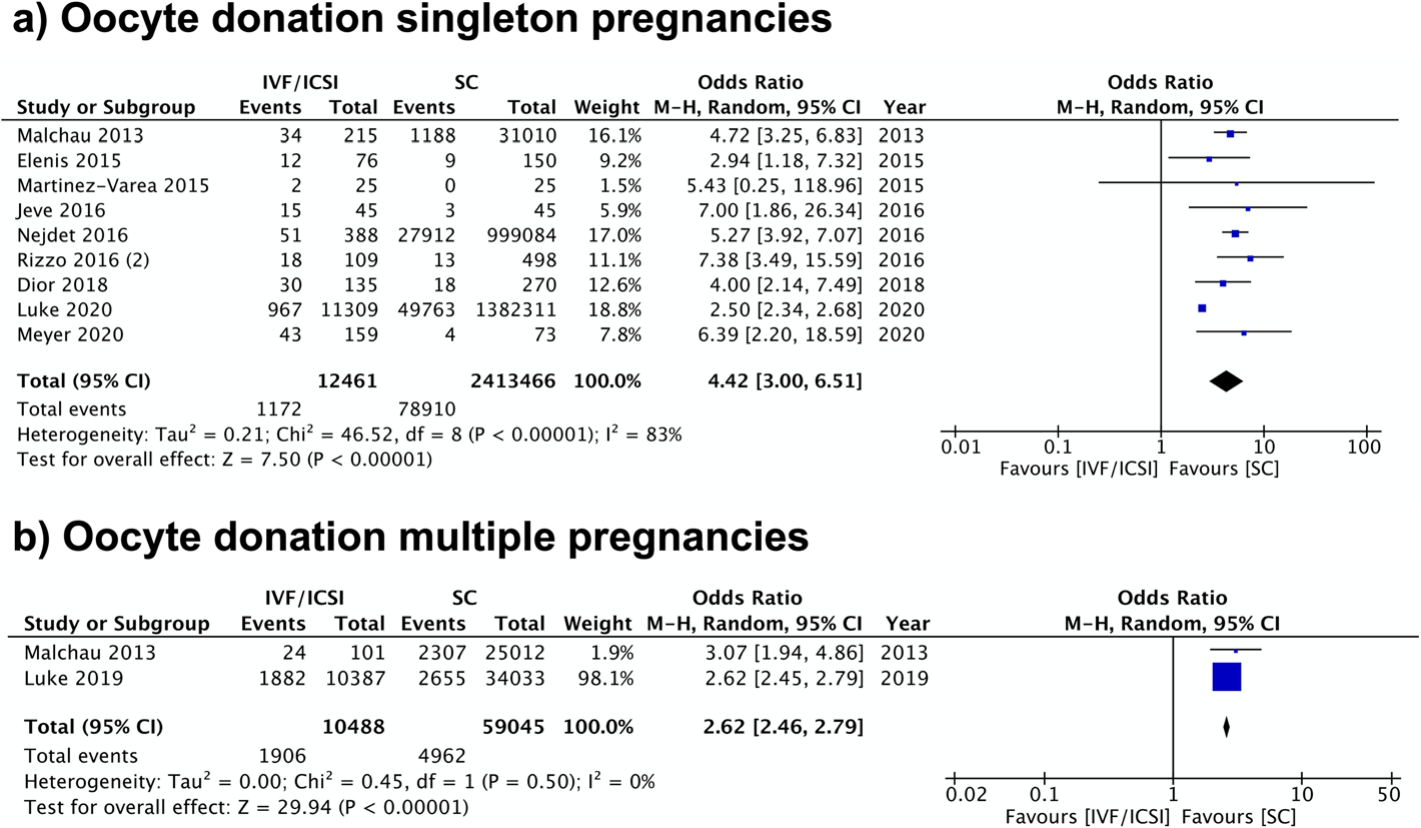
Oocyte Donation Meta-analysis. Forest plot comparing hypertensive disorders of pregnancy in **a**) singleton pregnancies or **b**) multiple pregnancies resulting from oocyte donation in comparison to spontaneous pregnancies

Pregnancies resulting from OD were found to have the highest risk of hypertensive complications of all analyses conducted for the study. For HDP, nine studies resulted in a pooled OR of 4.42 (95% CI 3.00–6.51; I^2^ = 83%) (Fig. 7). Similar findings were observed in multiple pregnancies, with an OR of 2.62 (95% CI 2.46–2.79) with no heterogeneity (I^2^ = 0%). However, only two studies were eligible (Fig. 7).

Seven studies studied preeclampsia as the outcome of interest. The resulting OR was 5.20 (95% CI 4.02–6.73) with low heterogeneity (I^2^ = 9%) (Additional file 5). All included studies had a moderate or high quality according to their NOS scores.

Overall, all IVF/ICSI groups were associated with increased odds of HDP in comparison to SC; however, the differences were insignificant when selected IVF and ICSI multiple pregnancies were analyzed separately. Similarly, all study groups except ICSI singleton pregnancies were associated with higher odds of preeclampsia. The difference between IVF multiple pregnancies and SC was small and insignificant. All findings described above were summarized in Table 2.

**Table 2:** Summary of results by type of ART and outcome of interest

Experimental	Number of studies	ART study size (n)	SC study size (n)	OR; 95% CI	I^2^ (%)	*P* value	NNH (n)
Hypertensive Disorders of Pregnancy							
IVF/ICSI singleton	51	268,166	7,728,641	1.70 (1.60–1.80)	80	< 0.01^a^	47.2
IVF singleton	5	41,238	437,723	1.55 (1.23–1.94)	90	< 0.01^a^	85.9
ICSI singleton	9	27,108	446,149	1.52 (1.28–1.80)	75	< 0.01^a^	54.3
IVF/ICSI multiple	41	88,853	120,396	1.34 (1.20–1.50)	76	< 0.01^a^	47.8
IVF multiple	4	13,147	73,297	1.13 (0.98–1.29)	59	0.09	86.3
ICSI multiple	10	9924	77,179	1.11 (0.91–1.36)	77	0.30	12.8
Fresh embryo transfer singleton	16	162,867	4,381,981	1.43 (1.33–1.53)	72	< 0.01^a^	57.4
Frozen embryo transfer singleton	9	41,462	4,090,152	1.74 (1.58–1.92)	55	< 0.01^a^	52.6
Oocyte donation singleton	9	12,461	2,413,466	4.42 (3.00–6.51)	83	< 0.01^a^	16.3
Oocyte donation multiple	2	10,488	59,045	2.62 (2.46–2.79)	0	< 0.01^a^	10.2
Preeclampsia							
IVF/ICSI singleton	28	118,787	4,509,905	1.59 (1.46–1.74)	70	< 0.01^a^	81.0
ICSI singleton	2	5807	31,124	0.98 (0.38–2.51)	72	0.97	105.2
IVF/ICSI multiple	17	14,842	34,337	1.24 (1.08–1.43)	32	< 0.01^a^	91.7
IVF multiple	2	5696	25,998	1.04 (0.93–1.16)	0	0.47	364.0
ICSI multiple	5	5618	29,519	1.11 (1.00–1.24)	0	0.05^a^	52.1
Fresh embryo transfer singleton	8	55,300	2,715,647	1.48 (1.37–1.60)	39	< 0.01^a^	85.5
Frozen embryo transfer singleton	5	19,216	2,699,693	1.82 (1.71–1.95)	0	< 0.01^a^	45.6
Oocyte donation singleton	7	1107	1,031,110	5.20 (4.02–6.73)	9	< 0.01^a^	10.1

^a^= Statistically significant. *NNH* Numbers needed to harm

### Numbers needed to harm

The numbers needed to harm for each IVF/ICSI subgroups were shown in Table 2. While most interventions had an NNH of 40 to 100, OD pregnancies had particularly low NNH. Sixteen patients who achieved singleton pregnancies through OD were needed to have one case of HDP; similarly, only ten multiple pregnancy patients through OD were needed to have one case of HDP.

### Sensitivity analysis and publication bias

Sensitivity analyses were performed to identify individual studies with large influences on the overall risk estimates. Exclusion of any study did not yield significantly different OR, with the exception of one study by Malchau et al. for the analysis of preeclampsia in IVF multiple pregnancies (Additional file 6). Funnel plots of meta-analyses involving more than 10 studies did not reveal any publication bias (Additional file 5).

## Discussion

### Principle findings

IVF/ICSI pregnancies, when compared to SC, carried higher odds of HDP and preeclampsia regardless of their plurality. While both fresh ET and FET were found to have odds of hypertensive complications, FET was associated with higher odds in comparison fresh ET. Pregnancies resulting from OD had the highest odds of HDP and preeclampsia out of all the groups analyzed.

Analyses on IVF and ICSI pregnancies specifically yielded mixed results and were limited by a relatively small number of studies. Although IVF pregnancies had higher incidences of HDP in comparison to SC, the difference was not statistically significant for multiple pregnancies. While ICSI singleton pregnancies resulted in higher odds of HDP compared to SC, the odds of preeclampsia were similar in both groups. In multiple pregnancy, although ICSI was associated with increased odds of preeclampsia, the difference with SC was small.

### Comparison with existing literature

Our results of higher odds of HDP and preeclampsia in singleton pregnancies following IVF/ICSI were in accordance with other previous meta-analyses [7, 10, 13, 14]. The measures of association for HDP in IVF/ICSI pregnancies (OR 1.70; 95% CI 1.60–1.80) was comparable to meta-analyses by Panday et al. (RR 1.49; 95% CI 1.39–1.59) and Qin et al. (RR 1.30; 95% CI 1.04–1.62) with a significant increase in statistical power due to a high number of studies included (51 studies) [7, 14]. Although the most recent meta-analysis by Thomopoulos et al. did not report data for IVF/ICSI pregnancies, the estimated relative risk for HDP in IVF pregnancies (RR 1.45; 95% CI 1.26–1.68) was similar to our study result (RR 1.55; 95% CI 1.23–1.94) [10]. This comprehensive systemic review included some cohort studies with mixed gestational orders, which was a strict exclusion criterion for our meta-analysis to minimize confounding bias. Our study results could not be directly compared to those by Almasi-Hashiani et al. as the study also included fertility treatments other than IVF/ICSI in the exposure group [13].

Multiple pregnancy following IVF/ICSI was also found to be at higher odds for preeclampsia and HDP, although the differences were smaller. This was likely because HDP and preeclampsia are known have a higher prevalence in multiple pregnancies than singleton pregnancies, resulting in higher risks of hypertensive complications in both SC and IVF/ICSI groups [68]. The meta-analysis by Qin et al. published in 2015 comprehensively studied the risk of HDP in IVF/ICSI multiple pregnancy. However, the study included other fertility treatments, such as ovulation induction and intrauterine insemination, in the control group, potentially underestimating the risk of IVF/ICSI on the outcome of interest. This might explain the slightly lower relative risk (RR 1.13; 95% CI 1.02–1.26) in comparison to our findings (OR 1.34; 95% CI 1.20–1.50) [9].

When studying ICSI pregnancies separately, our study found varying results for preeclampsia and HDP, and the findings were consistent with those reported by Thomopoulos and colleagues. While the meta-analysis by Thomopoulos et al. was limited by the small number of available studies, our sub-analysis included more recent studies and yielded similar findings [10]. Further research on ICSI pregnancies, as well as their indications for the procedure, such as male infertility, should be conducted to identify potential contributing factors.

Although the mechanism by which IVF/ICSI increases the risk of preeclampsia remains unknown, there exists a great body of literature studying potential causes. First, baseline maternal characteristics such as advanced maternal age, obesity, and medical comorbidities vary between the exposure and control groups and have been shown to be associated with preeclampsia [38, 94]. The underlying infertility diagnosis may also lead to varying maternal and perinatal outcomes. A large-scale cohort study by Stern and colleagues found that women with infertility due to tubal factors and ovulation disorders may be at a particularly high risk of HDP in comparison to their respective SC groups with the same diagnoses [8]. Patients with endometriosis may carry a higher risk of HDP than the general population, although the risk is attenuated when specifically studying women with ART [109]. Together, these findings suggest that an individual’s risk of HDP must be evaluated based on the mode of conception, along with other patient factors and comorbidities.

The presence of significant differences in the outcomes between fresh ET, FET, and OD pregnancies highlighted the potential role of the procedure on the development of hypertensive complications. In recent years, the use of cryopreservation has expanded widely from women with medical indications (e.g. with medical conditions or treatments that impairs fertility) to social embryo and oocyte freezing, including women who prefer to defer childbearing and transgender people as a part of their medical transition process [110]. There is a growing interest in the “freeze-all” strategy as a result of its decreased incidence of ovarian hyperstimulation syndrome without compromising live birth rates [110]. While FET carries many unique advantages, it is still important to understand the associated perinatal outcomes with the procedure. Our review of all available literature showed that both fresh ET and FET were associated with increased risks of preeclampsia and HDP in comparison to SC; furthermore, the observed differences were greater in FET pregnancies than fresh ET pregnancies, which was consistent with earlier evidence [17, 111, 112]. It is worth noting that most past literature used fresh ET as a control group instead, making it difficult to directly compare the results between studies.

The observed difference between fresh ET and FET may be explained by the absence of a corpus luteum (CL), as suggested by five recently published cohort studies [44, 99, 113–115]. SC typically develop under the presence of one CL, while the number of CL for IVF pregnancies varies depending on the type of procedure. Fresh IVF cycles typically involve more than one CL, whereas frozen IVF cycles and OD are usually performed under programmed cycles with exogenous hormones in the absence of a CL [113, 115].

von Versen-Hoynck and colleagues performed a prospective cohort study showing that women who conceived without a CL had a higher risk of preeclampsia than women with one or more CL. In the same paper, further analysis also demonstrated that programmed FET pregnancies were associated with higher incidences of preeclampsia than natural FET pregnancies, which had a comparable risk as fresh IVF pregnancies [99]. This finding was again supported by Luke and colleagues [115]. These studies proposed that performing FET during a natural cycle or with supplementation of missing hormones, such as relaxin, may potentially reduce the risk of preeclampsia and HDP. However, this theory alone could not explain the increased risk in fresh ET in comparison to SC, suggesting that there may be other components of the procedure or unaccounted confounders that increased the risk of hypertensive complications. As fresh ET pregnancies were found to be associated with other perinatal complications such as low birth weight and small for gestational age, it is important to balance the risks and benefits with each patient’s health status when considering treatment options [116].

OD is becoming a common standard practice for patients with reproductive disorders, diminished ovarian reserve, or advanced maternal age due to its relatively high success rate and comparable live delivery rates in comparison to autologous IVF pregnancies [117, 118]. In our study, women who achieved singleton pregnancies from donated oocytes carried four- to five-fold odds of preeclampsia and HDP in comparison to women who achieved pregnancy through SC. Multiple pregnancies from OD also had higher odds, although the differences with spontaneous multiple pregnancies were smaller.

This finding was consistent with other previous systematic reviews and meta-analyses that used SC as the control group [5, 119, 120]. Pecks et al. reported the OR for HDP in OD pregnancies in comparison to SC to be 6.60 (95% CI 4.55–9.57). However, some included studies did not adjust for plurality, potentially leading to an overestimation due to the known risk of multiple pregnancy. Similarly, Masoudian et al. calculated an odds ratio of 4.34 (95% CI 3.10–6.06) with studies that included both singleton and multiple pregnancies. Storgaard et al. reported an odds ratio of 2.45 (95% CI 1.53–1.93) and an odds ratio of 2.95 (95% CI 2.29–3.76) for HDP and preeclampsia, respectively. Since the most recent literature, five other cohort studies were published and included in our analyses [42, 43, 53, 63, 66].

In addition to the potential role of CL on maternal circulation, the increased risk of preeclampsia and HDP observed in women who conceived via OD has also been suggested to be a result of a heighten immunologic response between the mother and the allogenic oocyte [121]. This was formed on the basis that normal placentation requires the development of immunologic tolerance of the mother and the fetus; studies reporting an increased risk of preeclampsia in primiparous women and after a change in paternity in multiparous women further support this immunologic theory [121, 122]. Lashley and colleagues found that among successful and uncomplicated OD pregnancies, there was a higher level of human leukocyte antigen (HLA) matching between mother and fetus in than expected by chance, suggesting the role of HLA gene in the development of preeclampsia [123]. However, it is still important to consider other patient factors such as advanced maternal age, which is very common in this patient population and may also play a role to the increased risk of hypertensive disorders.

### Strengths and limitations

Our findings were generally consistent with previous literature; however, this study also carries many distinctive strengths. This is the most up-to-date, large-scale meta-analysis (including 85 studies and 8.5 million pregnancies) studying the association between IVF/ICSI pregnancies and HDP. Strict inclusion and exclusion criteria were employed to focus solely on IVF/ICSI pregnancies and SC (without any fertility treatment) to provide findings that are unique to this ART procedure. Recognizing the inherent risk of HDP in multiple pregnancies, all analyses stratified patients by plurality. Two outcomes, preeclampsia and HDP, were reported separately due to their differences in risk and prognosis. Furthermore, 89.4% of all included studies were of moderate to high quality (Table 1, Additional file 4). Finally, all publications in English, Chinese, Portuguese, and French were screened and reviewed to minimize language bias.

However, there were also some limitations. IVF-specific analyses were limited by the low number of studies that explicitly excluded ICSI pregnancies. The diagnostic criteria of preeclampsia differ depending on the study period and geographical location [75]. The lack of definition for the outcomes of interest among the cohorts (43% of all included studies) also made it difficult to create a uniform definition for the meta-analysis. This issue was solved by considering the following criteria: if a study used preeclampsia as an outcome without specifying the definition, it was included in analyses for preeclampsia. If a study used terminologies such as “pregnancy-induced hypertension”, “gestational hypertension”, or “hypertensive disorder”, it was only included in analyses for HDP. A high heterogeneity was reported in many pooled analyses, likely due to differences in study populations and geographical areas. Finally, uncontrolled confounders remained to be a concern due to the nature of the study design and could also influence heterogeneity. Some of the included studies did employ a matching method when selecting control subjects, while other studies accounted for potential confounders such as age, parity, medical comorbidities, year of birth, socioeconomic status, ethnic origin, location, and cause of infertility.

## Conclusions and implications

There has been an increasing amount of literature studying the relationship between ART and pregnancy and perinatal outcomes over the past decade, but there is also a lack of clinical practice guidelines for women who conceived through ART [124]. Results of our meta-analyses confirmed that IVF/ICSI pregnancies were at high odds of preeclampsia and HDP than SC, irrespective of the plurality. In particular, the odds in FET and OD pregnancies were high. Further population-based research studying different IVF treatment protocols should be considered. Health care providers should be aware of these risks and develop specific care plans and interventions for pregnancies conceived by IVF/ICSI to decrease the incidence of hypertensive complications and subsequently the risks of maternal morbidity and mortality. For example, a systematic review of randomized controlled trials by Henderson et al. suggested that daily low-dose aspirin starting after the first trimester might reduce the risk of preeclampsia [125]. The relationship between preeclampsia and ICSI singleton pregnancies remains unclear due to insufficient literature that studies this population. Given that the use of ICSI is gaining popularity over time, more research studying the pregnancy outcomes after ICSI is warranted.

## Supplementary Information

**Additional file 1.** Preferred Reporting Items for Systematic Reviews and Meta-Analyses (PRISMA) checklist. A list of 27 items required under the PRISMA statement with their respective locations.

**Additional file 2.** Search strategy for the systematic review and meta-analysis. Complete Search Strategy for a) Embase (1947 to 2020 April 08) b) Ovid MEDLINE, MEDLINE Daily and Epub Ahead of Print, In-Process & Other Non-Indexed Citations (1947 to 2020 April 08) c) EBM Reviews - Cochrane Central Register of Controlled Trials (1947 to April 2020).

**Additional file 3.** Excluded full text studies, with reasons. A list of full text studies excluded after screening with reasons for removal.

**Additional file 4.** Newcastle-Ottawa Scale for quality assessment and publication bias. A breakdown of NOS scores assigned to each study included in the meta-analysis.

**Additional file 5.** Forest plots for preeclampsia and funnel plots. Forest plots comparing preeclampsia in IVF/ICSI pregnancies and spontaneous pregnancies and funnel plots for publication bias in meta-analyses with 10 or more studies.

**Additional file 6.** Sensitivity analysis. A list of highest and lowest overall odds ratios after removing individual studies.

## Data Availability

The data underlying this article will be shared on reasonable request to the corresponding author.

## Abbreviations

ART: Assisted reproductive technologies
IVF: In vitro fertilization
ICSI: Intracytoplasmic sperm injection
Fresh ET: Fresh embryo transfer
FET: Frozen embryo transfer
OD: Oocyte donation
HDP: Hypertensive disorders of pregnancy
SC: Spontaneous conception
PRISMA: Preferred Reporting Items for Systematic Reviews and Meta-analyses
NOS: Newcastle Ottawa Scale
OR: Odds ratio
CL: Corpus luteum
HLA: Human leukocyte antigen
