# Supplementary material for: Assisted reproductive technology and hypertensive disorders of pregnancy: systematic review and meta-analyses
Source: BMC Pregnancy Childbirth. 2021 Jun 28;21:449. doi: 10.1186/s12884-021-03938-8 (PMC8240295; doi:10.1186/s12884-021-03938-8)
Supplement: Supplementary file 2 — Additional file 2. Search strategy for the systematic review and meta-analysis. Complete Search Strategy for a) Embase (1947 to 2020 April 08) b) Ovid MEDLINE, MEDLINE Daily and Epub Ahead of Print, In-Process & Other Non-Indexed Citations (1947 to 2020 April 08) c) EBM Reviews - Cochrane Central Register of Controlled Trials (1947 to April 2020). [file 12884_2021_3938_MOESM2_ESM.docx]

Additional file 2. Search strategy for the systematic review and meta-analysis.

| **#** | **Searches** | **Results** |
| --- | --- | --- |
| 1 | infertility therapy/ or exp in vitro fertilization/ | 84059 |
| 2 | (assist* adj3 (conceiv* or concepti* or reproducti*)).tw,kw. | 26416 |
| 3 | (reproducti* adj3 (technic or technics or technique*)).tw,kw. | 5823 |
| 4 | ART.tw,kw. | 131608 |
| 5 | ("In Vitro" adj3 (fertili* or reproducti*)).tw,kw. | 36059 |
| 6 | IVF.tw,kw. | 41846 |
| 7 | intracytoplasmic sperm injection*.tw,kw. | 9846 |
| 8 | intra-cytoplasmic sperm injection*.tw,kw. | 630 |
| 9 | 1 or 2 or 3 or 4 or 5 or 6 or 7 or 8 | 237032 |
| 10 | maternal hypertension/ | 18275 |
| 11 | exp "eclampsia and preeclampsia"/ | 64310 |
| 12 | ((maternal or gestational or pregnan*) adj3 hypertens*).tw,kw. | 22744 |
| 13 | preeclampsia.tw,kw. | 34808 |
| 14 | pre-eclampsia.tw,kw. | 16403 |
| 15 | eclampsia.tw,kw. | 23930 |
| 16 | 10 or 11 or 12 or 13 or 14 or 15 | 84711 |
| 17 | 9 and 16 | 2406 |
| 18 | limit 17 to conference abstract | 701 |
| 19 | 17 not 18 | 1705 |
| 20 | limit 19 to "review" | 374 |
| 21 | 19 not 20 | 1331 |

| **#** | **Searches** | **Results** |
| --- | --- | --- |
| 1 | reproductive techniques, assisted/ or fertilization in vitro/ or sperm injections, intracytoplasmic/ | 42961 |
| 2 | (assist* adj3 (conceiv* or concepti* or reproducti*)).tw,kw. | 16153 |
| 3 | (reproducti* adj3 (technic or technics or technique*)).tw,kw. | 3213 |
| 4 | ART.tw,kw. | 101233 |
| 5 | ("In Vitro" adj3 (fertili* or reproducti*)).tw,kw. | 26041 |
| 6 | IVF.tw,kw. | 23795 |
| 7 | intracytoplasmic sperm injection*.tw,kw. | 7217 |
| 8 | intra-cytoplasmic sperm injection*.tw,kw. | 314 |
| 9 | 1 or 2 or 3 or 4 or 5 or 6 or 7 or 8 | 159112 |
| 10 | hypertension, pregnancy-induced/ or eclampsia/ or hellp syndrome/ or pre-eclampsia/ | 36115 |
| 11 | ((maternal or gestational or pregnan*) adj3 hypertens*).tw,kw. | 15283 |
| 12 | preeclampsia.tw,kw. | 21147 |
| 13 | pre-eclampsia.tw,kw. | 10552 |
| 14 | eclampsia.tw,kw. | 15473 |
| 15 | 10 or 11 or 12 or 13 or 14 | 55127 |
| 16 | 9 and 15 | 883 |
| 17 | limit 16 to "review articles" | 182 |
| 18 | 16 not 17 | 701 |

| **#** | **Searches** | **Results** |
| --- | --- | --- |
| 1 | reproductive techniques, assisted/ or fertilization in vitro/ or sperm injections, intracytoplasmic/ | 2138 |
| 2 | (assist* adj3 (conceiv* or concepti* or reproducti*)).tw,kw. | 1711 |
| 3 | (reproducti* adj3 (technic or technics or technique*)).tw,kw. | 339 |
| 4 | ART.tw,kw. | 7107 |
| 5 | ("In Vitro" adj3 (fertili* or reproducti*)).tw,kw. | 3733 |
| 6 | IVF.tw,kw. | 5890 |
| 7 | intracytoplasmic sperm injection*.tw,kw. | 1901 |
| 8 | intra-cytoplasmic sperm injection*.tw,kw. | 121 |
| 9 | 1 or 2 or 3 or 4 or 5 or 6 or 7 or 8 | 14960 |
| 10 | hypertension, pregnancy-induced/ or eclampsia/ or hellp syndrome/ or pre-eclampsia/ | 1036 |
| 11 | ((maternal or gestational or pregnan*) adj3 hypertens*).tw,kw. | 2075 |
| 12 | preeclampsia.tw,kw. | 2480 |
| 13 | pre-eclampsia.tw,kw. | 1100 |
| 14 | eclampsia.tw,kw. | 1469 |
| 15 | 10 or 11 or 12 or 13 or 14 | 4584 |
| 16 | 9 and 15 | 84 |
