# Supplementary material for: Assisted reproductive technology and hypertensive disorders of pregnancy: systematic review and meta-analyses
Source: BMC Pregnancy Childbirth. 2021 Jun 28;21:449. doi: 10.1186/s12884-021-03938-8 (PMC8240295; doi:10.1186/s12884-021-03938-8)
Supplement: Supplementary file 3 — Additional file 3. Excluded full text studies, with reasons. A list of full text studies excluded after screening with reasons for removal. [file 12884_2021_3938_MOESM3_ESM.docx]

Additional file 3. List of excluded studies after full-text screening, with reasons.

Does not clearly separate data/combined data.

1. Erez O, Vardi IS, Hallak M, Hershkovitz R, Dukler D, Mazor M. Preeclampsia in twin gestations: association with IVF treatments, parity and maternal age. The journal of maternal-fetal & neonatal medicine : the official journal of the European Association of Perinatal Medicine, the Federation of Asia and Oceania Perinatal Societies, the International Society of Perinatal Obstetricians 2006;19: 141-146.
2. Kallen B, Finnstrom O, Nygren KG, Otterblad Olausson P, Wennerholm U-B. In vitro fertilisation in Sweden: obstetric characteristics, maternal morbidity and mortality. BJOG : an international journal of obstetrics and gynaecology 2005;112: 1529-1535.
3. Khatibi A, Nybo Andersen A-M, Gissler M, Morken N-H, Jacobsson B. Obstetric and neonatal outcome in women aged 50 years and up: A collaborative, Nordic population-based study. European journal of obstetrics, gynecology, and reproductive biology 2018;224: 17-20.
4. Roos N, Kieler H, Sahlin L, Ekman-Ordeberg G, Falconer H, Stephansson O. Risk of adverse pregnancy outcomes in women with polycystic ovary syndrome: population based cohort study. BMJ (Clinical research ed) 2011;343: d6309.
5. Sanchez O, Llurba E, Marsal G, Dominguez C, Aulesa C, Sanchez-Duran MA, Goya MM, Alijotas-Reig J, Carreras E, Cabero L. First trimester serum angiogenic/anti-angiogenic status in twin pregnancies: relationship with assisted reproduction technology. Human reproduction (Oxford, England) 2012;27: 358-365.
6. Stephansson O, Kieler H, Granath F, Falconer H. Endometriosis, assisted reproduction technology, and risk of adverse pregnancy outcome. Human reproduction (Oxford, England) 2009;24: 2341-2347.
7. Tabs D, Vejnovic T, Radunovic N. Preeclampsia and eclampsia in parturients from the in vitro fertilization program. Medicinski pregled 2004;57: 7-12.
8. Udholm S, Udholm L, Nyboe C, Kesmodel US, Hjortdal VE. Pregnancy outcome in women with atrial septal defect: associated with in vitro fertilisation and pre-eclampsia. Open heart 2019;6: e001148.
9. van Zijl MD, Koullali B, Oudijk MA, Ravelli ACJ, Mol BWJ, Pajkrt E, Kazemier BM. Trends in preterm birth in singleton and multiple gestations in the Netherlands 2008-2015: A population-based study. European Journal of Obstetrics and Gynecology and Reproductive Biology 2020;247: 111-115.
10. Rendtorff R, Hinkson L, Kiver V, Droge LA, Henrich W. Pregnancies in Women Aged 45 Years and Older - a 10-Year Retrospective Analysis in Berlin. Geburtshilfe und Frauenheilkunde 2017;77: 268-275.
11. Lamminpaa R, Vehvilainen-Julkunen K, Gissler M, Heinonen S. Preeclampsia complicated by advanced maternal age: a registry-based study on primiparous women in Finland 1997-2008. BMC pregnancy and childbirth 2012;12: 47.

Does not clearly separate patients into singleton pregnancy and non-singleton pregnancy groups.

1. Bay B, Ingerslev HJ, Lemmen JG, Degn B, Rasmussen IA, Kesmodel US. Preimplantation genetic diagnosis: a national multicenter obstetric and neonatal follow-up study. Fertility and sterility 2016;106: 1363-1369.e1361.
2. Ben-Yaakov RD, Kessous R, Shoham-Vardi I, Sergienko R, Pariente G, Sheiner E. Fertility Treatments in Women Who Become Pregnant and Carried to Viability, and the Risk for Long-Term Maternal Cardiovascular Morbidity. American journal of perinatology 2016;33: 1388-1393.
3. Jie Z, Yiling D, Ling Y. Association of assisted reproductive technology with adverse pregnancy outcomes. Iranian journal of reproductive medicine 2015;13: 169-180.
4. Sabban H, Zakhari A, Patenaude V, Tulandi T, Abenhaim HA. Obstetrical and perinatal morbidity and mortality among in-vitro fertilization pregnancies: a population-based study. Archives of gynecology and obstetrics 2017;296: 107-113.
5. Valenzuela-Alcaraz B, Crispi F, Manau D, Cruz-Lemini M, Borras A, Balasch J, Gratacos E. Differential effect of mode of conception and infertility treatment on fetal growth and prematurity. The journal of maternal-fetal & neonatal medicine : the official journal of the European Association of Perinatal Medicine, the Federation of Asia and Oceania Perinatal Societies, the International Society of Perinatal Obstetricians 2016;29: 3879-3884.
6. Wang ET, Ozimek JA, Greene N, Ramos L, Vyas N, Kilpatrick SJ, Pisarska MD. Impact of fertility treatment on severe maternal morbidity. Fertility and sterility 2016;106: 423-426.
7. Wang Q-Q, Zhu Y-M, Wu M-Y. [Health status of mothers undergoing in vitro fertilization and their offspring]. Zhejiang da xue xue bao Yi xue ban = Journal of Zhejiang University Medical sciences 2009;38: 515-520.
8. Ye R, Zhang L, Yang Z. [Obstetrical outcome of pregnancy on in vitro fertilization and embryo transfer 128 cases analysis]. Zhonghua fu chan ke za zhi 2000;35: 157-159.
9. Johnston R, Fong A, Lovell S, Sobolewski PS, Rad S, Turner A. Demographic and Obstetric Outcomes of Pregnancies conceived by Assisted Reproductive Technology (ART) compared to Non-ART Pregnancies. JBRA assisted reproduction 2015;19: 16-20.
10. Miyake H, Iwasaki N, Nakai A, Suzuki S, Takeshita T. The influence of assisted reproductive technology on women with pregnancy-induced hypertension: a retrospective study at a Japanese Regional Perinatal Center. Journal of Nippon Medical School = Nippon Ika Daigaku zasshi 2010;77: 312-317.
11. Marchand E, Poncelet C, Carbillon L, Pharisien I, Tigaizin A, Chanelles O. [Is there more complications with pregnancies from the assisted reproductive technology than spontaneous pregnancies? A retrospective study over 6 years]. Journal de gynecologie, obstetrique et biologie de la reproduction 2011;40: 522-528.
12. Gui J, Ling Z, Hou X, Fan Y, Xie K, Shen R. In vitro fertilization is associated with the onset and progression of preeclampsia. *Placenta* 2020;89: 50-57.

Does not have spontaneous pregnancies as the control group.

1. Lang J, Zhang B, Meng Y, Du Y, Cui L, Li W. First trimester depression and/or anxiety disorders increase the risk of low birthweight in IVF offspring: a prospective cohort study. Reproductive biomedicine online 2019;39: 947-954.
2. Laskov I, Birnbaum R, Maslovitz S, Kupferminc M, Lessing J, Many A. Outcome of singleton pregnancy in women >=45 years old: A retrospective cohort study. Journal of Maternal-Fetal and Neonatal Medicine 2012;25: 2190-2193.
3. Letur H, Peigne M, Ohl J, Cedrin-Durnerin I, Mathieu-D'Argent E, Scheffler F, Grzegorczyk-Martin V, de Mouzon J. Hypertensive pathologies and egg donation pregnancies: Results of a large comparative cohort study. Fertility and sterility 2016;106: 284-290.
4. Liu L, Wang H, Zhang Y, Niu J, Li Z, Tang R. Effect of pregravid obesity on perinatal outcomes in singleton pregnancies following in vitro fertilization and the weight-loss goals to reduce the risks of poor pregnancy outcomes: A retrospective cohort study. PloS one 2020;15: e0227766.
5. Liu SY, Teng B, Fu J, Li X, Zheng Y, Sun XX. Obstetric and neonatal outcomes after transfer of vitrified early cleavage embryos. Human reproduction (Oxford, England) 2013;28: 2093-2100.
6. Luke B, Stern JE, Kotelchuck M, Declercq ER, Anderka M, Diop H. Birth Outcomes by Infertility Treatment: Analyses of the Population-Based Cohort: Massachusetts Outcomes Study of Assisted Reproductive Technologies (MOSART). The Journal of reproductive medicine 2016;61: 114-127.
7. Magnusson A, Wennerholm U-B, Kallen K, Petzold M, Thurin-Kjellberg A, Bergh C. The association between the number of oocytes retrieved for IVF, perinatal outcome and obstetric complications. Human reproduction (Oxford, England) 2018;33: 1939-1947.
8. Oron G, Hiersch L, Rona S, Prag-Rosenberg R, Sapir O, Tuttnauer-Hamburger M, Shufaro Y, Fisch B, Ben-Haroush A. Endometrial thickness of less than 7.5 mm is associated with obstetric complications in fresh IVF cycles: a retrospective cohort study. Reproductive biomedicine online 2018;37: 341-348.
9. Oron G, Sokal-Arnon T, Son W-Y, Demirtas E, Buckett W, Zeadna A, Holzer H, Tulandi T. Extended embryo culture is not associated with increased adverse obstetric or perinatal outcome. American journal of obstetrics and gynecology 2014;211: 165.e161-167.
10. Pinborg A, Lidegaard O, Freiesleben NlC, Andersen AN. Vanishing twins: a predictor of small-for-gestational age in IVF singletons. Human reproduction (Oxford, England) 2007;22: 2707-2714.
11. Pinborg A, Loft A, Schmidt L, Langhoff-Roos J, Andersen AN. Maternal risks and perinatal outcome in a Danish national cohort of 1005 twin pregnancies: the role of in vitro fertilization. Acta obstetricia et gynecologica Scandinavica 2004;83: 75-84.
12. Rahu K, Allvee K, Karro H, Rahu M. Singleton pregnancies after in vitro fertilization in Estonia: a register-based study of complications and adverse outcomes in relation to the maternal socio-demographic background. BMC pregnancy and childbirth 2019;19: 51.
13. Saito K, Kuwahara A, Ishikawa T, Morisaki N, Miyado M, Miyado K, Fukami M, Miyasaka N, Ishihara O, Irahara M et al. Endometrial preparation methods for frozen-thawed embryo transfer are associated with altered risks of hypertensive disorders of pregnancy, placenta accreta, and gestational diabetes mellitus. Human reproduction (Oxford, England) 2019;34: 1567-1575.
14. Shavit T, Oron G, Weon-Young S, Holzer H, Tulandi T. Vitrified-warmed single-embryo transfers may be associated with increased maternal complications compared with fresh single-embryo transfers. Reproductive BioMedicine Online 2017;35: 94-102.
15. Sites CK, Wilson D, Barsky M, Bernson D, Bernstein IM, Boulet S, Zhang Y. Embryo cryopreservation and preeclampsia risk. Fertility and sterility 2017;108: 784-790.
16. Suzuki S, Miyake H. Obstetric outcomes in nulliparous women aged 35 and over with singleton pregnancies conceived by in vitro fertilization. Archives of gynecology and obstetrics 2008;277: 225-227.
17. Tsutsumi R, Fujimoto A, Osuga Y, Ooi N, Takemura Y, Koizumi M, Yano T, Taketani Y. Singleton pregnancy outcomes after assisted and non-assisted reproductive technology in infertile patients. Reproductive medicine and biology 2012;11: 149-153.
18. Uysal NS, Gulumser C, Bilgin Yanik FF, Onalan G, Zeyneloglu HB. Outcome of ICSI pregnancies with spontaneous loss of a co-twin compared with singleton ICSI pregnancies: Single center experience. The journal of obstetrics and gynaecology research 2020;46: 445-450.
19. Wang Y, Li H, Li L, Wang X. Study on fetal reduction in multiple births. International Journal of Clinical and Experimental Medicine 2016;9: 6846-6849.
20. Wang Y, Zhao X, Zhao H, Ding H, Tan J, Chen J, Zhang R, Azziz R, Yang D. Risks for gestational diabetes mellitus and pregnancy-induced hypertension are increased in polycystic ovary syndrome. BioMed research international 2013;2013: 182582.
21. Wise LA, Mikkelsen EM, Sorensen HT, Rothman KJ, Hahn KA, Riis AH, Hatch EE. Prospective study of time to pregnancy and adverse birth outcomes. Fertility and Sterility 2015;103: 1065.
22. Yan X, Shi Y-h, Sheng Y, Tang R, Xu L-h, Li Y, Chen Z-j. [Pregnancy outcomes of patients with polycystic ovary syndrome undergoing in vitro fertilization and embryo transfer]. Zhonghua fu chan ke za zhi 2011;46: 923-927.
23. De Vos A, Janssens R, Van de Velde H, Haentjens P, Bonduelle M, Tournaye H, Verheyen G. The type of culture medium and the duration of in vitro culture do not influence birthweight of ART singletons. Human reproduction (Oxford, England) 2015;30: 20-27.
24. De Vos A, Santos-Ribeiro S, Van Landuyt L, Van de Velde H, Tournaye H, Verheyen G. Birthweight of singletons born after cleavage-stage or blastocyst transfer in fresh and warming cycles. Human reproduction (Oxford, England) 2018;33: 196-201.
25. Fernando D, Halliday JL, Breheny S, Healy DL. Outcomes of singleton births after blastocyst versus nonblastocyst transfer in assisted reproductive technology. Fertility and sterility 2012;97: 579-584.
26. Ishihara O, Araki R, Kuwahara A, Itakura A, Saito H, Adamson GD. Impact of frozen-thawed single-blastocyst transfer on maternal and neonatal outcome: an analysis of 277,042 single-embryo transfer cycles from 2008 to 2010 in Japan. Fertility and sterility 2014;101: 128-133.
27. Kennedy AL, Stern CJ, Tong S, Hastie R, Agresta F, Walker SP, Brownfoot FC, MacLachlan V, Vollenhoven BJ, Lindquist AC. The incidence of hypertensive disorders of pregnancy following sperm donation in IVF: an Australian state-wide retrospective cohort study. Human reproduction (Oxford, England) 2019;34: 2541-2548.
28. Moaddab A, Chervenak FA, McCullough LB, Sangi-Haghpeykar H, Shamshirsaz AA, Schutt A, Arian SE, Fox KA, Dildy GA, Shamshirsaz AA. Effect of advanced maternal age on maternal and neonatal outcomes in assisted reproductive technology pregnancies. European journal of obstetrics, gynecology, and reproductive biology 2017;216: 178-183.
29. Sazonova A, Kallen K, Thurin-Kjellberg A, Wennerholm U-B, Bergh C. Neonatal and maternal outcomes comparing women undergoing two in vitro fertilization (IVF) singleton pregnancies and women undergoing one IVF twin pregnancy. Fertility and sterility 2013;99: 731-737.
30. Chasen ST, Luo G, Perni SC, Kalish RB. Are in vitro fertilization pregnancies with early spontaneous reduction high risk? American journal of obstetrics and gynecology 2006;195: 814-817.
31. Sterling L, Liu J, Okun N, Sakhuja A, Sierra S, Greenblatt E. Pregnancy outcomes in women with polycystic ovary syndrome undergoing in vitro fertilization. Fertility and sterility 2016;105: 791-797.e792.
32. Wan HLT, Hui PW, Li HWR, Ng EHY. Obstetric outcomes in women with polycystic ovary syndrome and isolated polycystic ovaries undergoing in vitro fertilization: a retrospective cohort analysis. The journal of maternal-fetal & neonatal medicine : the official journal of the European Association of Perinatal Medicine, the Federation of Asia and Oceania Perinatal Societies, the International Society of Perinatal Obstetricians 2015;28: 475-478.
33. Cassell KA, O'Connell CM, Baskett TF. The origins and outcomes of triplet and quadruplet pregnancies in Nova Scotia: 1980 to 2001. American Journal of Perinatology 2004;21: 439-445.
34. Ma J, Sun XX, Wu Y, Gu W, Li L. Effects of ovarian poor response on incidences of hypertensive disorders complicating pregnancy after in vitro fertilization. Journal of Shanghai Jiaotong University (Medical Science) 2011;31: 967-970.
35. Tarlatzi TB, Imbert R, Alvaro Mercadal B, Demeestere I, Venetis CA, Englert Y, Delbaere A. Does oocyte donation compared with autologous oocyte IVF pregnancies have a higher risk of preeclampsia? Reproductive biomedicine online 2017;34: 11-18.
36. Le Ray C, Scherier S, Anselem O, Marszalek A, Tsatsaris V, Cabrol D, Goffinet F. Association between oocyte donation and maternal and perinatal outcomes in women aged 43 years or older. Human reproduction (Oxford, England) 2012;27: 896-901.
37. Ulkumen B, Silfeler D, Sofuoglu K, Silfeler I, Dayicioglu V. The incidence of preeclampsia in Intra-Cytoplasmic Sperm Injection pregnancies. Pakistan Journal of Medical Sciences 2013;30.
38. Tanaka H, Tanaka K, Osato K, Kusaka H, Maegawa Y, Taniguchi H, Ikeda T. Evaluation of Maternal and Neonatal Outcomes of Assisted Reproduction Technology: A Retrospective Cohort Study. Medicina (Kaunas, Lithuania) 2020;56.
39. Woo I, Hindoyan R, Landay M, Ho J, Ingles SA, McGinnis LK, Paulson RJ, Chung K. Perinatal outcomes after natural conception versus in vitro fertilization (IVF) in gestational surrogates: a model to evaluate IVF treatment versus maternal effects. Fertility and sterility 2017;108: 993-998.
40. Lucovnik M, Blickstein I, Mirkovic T, Verdenik I, Bricelj K, Vidmar Simic M, Tul N, Trojner Bregar A. Effect of pre-gravid body mass index on outcomes of pregnancies following in vitro fertilization. Journal of assisted reproduction and genetics 2018;35: 1309-1315.
41. Carassou-Maillan A, Pouly JL, Mulliez A, Dejou-Bouillet L, Gremeau AS, Brugnon F, Janny L, Canis M. [Adverse pregnancy outcomes after Assisted Reproduction Technology in women with endometriosis]. Gynecologie, obstetrique & fertilite 2014;42: 210-215.
42. Carmona-Ruiz IO, Saucedo de la Llata E, Moraga-Sanchez MR, Cantero-Minano MD, Romeu-Sarrio A. Gestational weight gain and perinatal outcomes: Study of a Spanish population and the effect of assisted reproduction techniques. Ginecologia y Obstetricia de Mexico 2016;84: 684-695.
43. Guilbaud L, Santulli P, Studer E, Gayet V, Goffinet F, Le Ray C. Impact of oocyte donation on perinatal outcome in twin pregnancies. Fertility and sterility 2017;107: 948-953.e941.

Does not include ART as exposure group.

1. Dey M, Saraswat M. Outcomes of Multifetal Reduction: A Hospital-Based Study. Journal of obstetrics and gynaecology of India 2018;68: 264-269.
2. Dildy GA, Jackson GM, Fowers GK, Oshiro BT, Varner MW, Clark SL. Very advanced maternal age: pregnancy after age 45. American journal of obstetrics and gynecology 1996;175: 668-674.
3. Shand AW, Whitton K, Pasfield A, Nassar N, McShane M, Han X, Henry A. Evaluation of anti-Mullerian hormone in the first trimester as a predictor for hypertensive disorders of pregnancy and other adverse pregnancy outcomes. Australian and New Zealand Journal of Obstetrics and Gynaecology 2014;54: 244-249.
4. Takaoka S, Ishii K, Taguchi T, Kakubari R, Muto H, Mabuchi A, Yamamoto R, Hayashi S, Mitsuda N. Clinical features and antenatal risk factors for postpartum-onset hypertensive disorders. Hypertension in pregnancy 2016;35: 22-31.
5. Vieira LA, Warren L, Pan S, Ferrara L, Stone JL. Comparing pregnancy outcomes and loss rates in elective twin pregnancy reduction with ongoing twin gestations in a large contemporary cohort. American journal of obstetrics and gynecology 2019;221: 253.e251-253.e258.
6. Wallenius M, Salvesen KA, Daltveit AK, Skomsvoll JF. Systemic Lupus Erythematosus and Outcomes in First and Subsequent Births Based on Data From a National Birth Registry. Arthritis Care and Research 2014;66: 1718-1724.
7. T TZ, Weintraub AY, Gutman OA, Baumfeld Y, Soriano D, Mastrolia SA, E SH. Pregnancy outcomes in women with endometriosis. Minerva Ginecologica 2018;70: 144-149.
8. Zsirai L, Csakany GM, Vargha P, Fulop V, Tabak AG. Breech presentation: its predictors and consequences. An analysis of the Hungarian Tauffer Obstetric Database (1996-2011). Acta obstetricia et gynecologica Scandinavica 2016;95: 347-354.
9. Roberts CL, Algert CS, Morris JM, Ford JB. Increased planned delivery contributes to declining rates of pregnancy hypertension in Australia: a population-based record linkage study. BMJ open 2015;5: e009313.

Does not specify the fertility treatments included.

1. Wang AY, Safi N, Ali F, Lui K, Li Z, Umstad MP, Sullivan EA. Neonatal outcomes among twins following assisted reproductive technology: an Australian population-based retrospective cohort study. BMC pregnancy and childbirth 2018;18: 320.
2. Oberg AS, VanderWeele TJ, Almqvist C, Hernandez-Diaz S. Pregnancy complications following fertility treatment-disentangling the role of multiple gestation. International journal of epidemiology 2018;47: 1333-1342.
3. Shah JS, Roman T, Viteri OA, Haidar ZA, Ontiveros A, Sibai BM. The Relationship of Assisted Reproductive Technology on Perinatal Outcomes in Triplet Gestations. American journal of perinatology 2018;35: 1388-1393.

Duplicate.

1. Oron G, Son W-Y, Buckett W, Tulandi T, Holzer H. The association between embryo quality and perinatal outcome of singletons born after single embryo transfers: a pilot study. Human reproduction (Oxford, England) 2014;29: 1444-1451.
2. Ulkumen B, Silfeler D, Sofuoglu K, Silfeler I, Dayicioglu V. The incidence of preeclampsia in intra-Cytoplasmic Sperm injection pregnancies. Pakistan Journal of Medical Sciences 2014;30: 101-105.
3. Ginstrom Ernstad E, Bergh C, Khatibi A, Kallen KBM, Westlander G, Nilsson S, Wennerholm UB. Neonatal and maternal outcome after blastocyst transfer: A population-based registry study Presented orally at the 36th Society for Maternal-Fetal Medicine Annual Pregnancy Meeting, Atlanta, GA, Feb. 1-6, 2016. American Journal of Obstetrics and Gynecology 2016;214: 378.
4. Montoya JB, Munoz ER, Rivera EC, Villasenor BL, Diaz JFJ, Canedo TH. Adverse perinatal outcomes in Mexican women with pregnancies assisted reproduction twin vs twin spontaneous. Ginecologia y Obstetricia de Mexico 2012;80: 445-453.
5. Okun N, Sierra S, Genetics C, Special C. Pregnancy outcomes after assisted human reproduction. Journal of obstetrics and gynaecology Canada : JOGC = Journal d'obstetrique et gynecologie du Canada : JOGC 2014;36: 64-83.
6. Qin J, Sheng X, Wu D, Gao S, You Y, Yang T, Wang H. Adverse Obstetric Outcomes Associated With In Vitro Fertilization in Singleton Pregnancies: A Prospective Cohort Study. Reproductive sciences (Thousand Oaks, Calif) 2017;24: 595-608.

Full text not available.

1. Brubel R, Dobo N, Csibi N, Kovesdi A, Mate S, Acs N, Lukovich P, Murber A, Bokor A. [The effect of surgical treatment of bowel endometriosis on fertility]. Orvosi hetilap 2019;160: 1633-1638.
2. Chen Q, Ye H, Ding X, Shen X, Huang G, Deng H, Wen H, Pei L, Zeng P. A retrospective analysis of outcomes of 585 selectively reduced multiple pregnancies in IVF/ICSI-ET cycles. 2016;31: i288.
3. Sakai N, Kishimoto Y, Saito T, Ito M, Nakahara K, Saito H, Hiroi M. Perinatal risks after in vitro fertilization and embryo transfer. Japanese Journal of Fertility and Sterility 1996;41: 35-42.
4. Carmona F, Balasch J, Creus M, Fabregues F, Puerto B, Ballesca JL, Moreno V, Civico S, Cararach V, Vanrell JA. Obstetric and perinatal outcome in pregnancies obtained through IVF. *Progresos en Obstetricia y Ginecologia* 1994;37: 219-225.

Included ART other than IVF and ICSI.

1. DoPierala AL, Bhatta S, Raja EA, Bhattacharya S. Obstetric consequences of subfertility: a retrospective cohort study. BJOG: An International Journal of Obstetrics and Gynaecology 2016;123: 1320-1328.
2. Park HS, Kwon H, McElrath FF. Assisted reproductive technology and the risk of unplanned peripartum hysterectomy: Analysis using propensity score matching. Human Reproduction 2018;33: 1466-1473.
3. Shevell T, Malone FD, Vidaver J, Porter TF, Luthy DA, Comstock CH, Hankins GD, Eddleman K, Dolan S, Dugoff L et al. Assisted reproductive technology and pregnancy outcome. Obstetrics and gynecology 2005;106: 1039-1045.
4. Tanbo T, Dale PO, Lunde O, Moe N, Abyholm T. Obstetric outcome in singleton pregnancies after assisted reproduction. Obstetrics and gynecology 1995;86: 188-192.
5. Vulic M, Roje D, Mestrovic Z, Benzon Z, Miserda A, Kopic D. Is there a difference in incidence of preeclampsia in twin pregnancies concieved spontaneously and after assisted conception. Gynaecologia et Perinatologia 2014;23: 49-53.
6. Wang YA, Chughtai AA, Farquhar CM, Pollock W, Lui K, Sullivan EA. Increased incidence of gestational hypertension and preeclampsia after assisted reproductive technology treatment. Fertility and sterility 2016;105: 920-926.e922.
7. Wu Y, Chen Y, Shen M, Guo Y, Wen SW, Lanes A, White RR, Adanlawo A, Walker M, Hua X. Adverse maternal and neonatal outcomes among singleton pregnancies in women of very advanced maternal age: a retrospective cohort study. BMC pregnancy and childbirth 2019;19: 3.
8. Zaib-un-Nisa S, Ghazal-Aswad S, Badrinath P. Outcome of twin pregnancies after assisted reproductive techniques--a comparative study. European journal of obstetrics, gynecology, and reproductive biology 2003;109: 51-54.
9. Fitzsimmons BP, Bebbington MW, Fluker MR. Perinatal and neonatal outcomes in multiple gestations: assisted reproduction versus spontaneous conception. American journal of obstetrics and gynecology 1998;179: 1162-1167.
10. Hurley EG, DeFranco EA. Influence of paternal age on perinatal outcomes. American journal of obstetrics and gynecology 2017;217: 566.e561-566.e566.
11. Tepper NK, Farr SL, Cohen BB, Nannini A, Zhang Z, Anderson JE, Jamieson DJ, Macaluso M. Singleton preterm birth: risk factors and association with assisted reproductive technology. Maternal and child health journal 2012;16: 807-813.
12. Schieve LA, Cohen B, Nannini A, Ferre C, Reynolds MA, Zhang Z, Jeng G, Macaluso M, Wright VC, Massachusetts Consortium for Assisted Reproductive Technology Epidemiologic R. A population-based study of maternal and perinatal outcomes associated with assisted reproductive technology in Massachusetts. Maternal and child health journal 2007;11: 517-525.
13. Declercq E, Luke B, Belanoff C, Cabral H, Diop H, Gopal D, Hoang L, Kotelchuck M, Stern JE, Hornstein MD. Perinatal outcomes associated with assisted reproductive technology: The Massachusetts Outcomes Study of Assisted Reproductive Technologies (MOSART). Fertility and Sterility 2015;103: 888-895.
14. Frati P, Foldes-Papp Z, Panici PB, Brunelli R, Zaami S, Busardo FP, Fineschi V. A Retrospective Study on Advanced Maternal Age and Assisted Reproductive Techniques, Medico-Legal Advice, "Food for Thought". Current pharmaceutical biotechnology 2016;17: 330-336.
15. Huang L-S, Yen C-H, Lee S-H, Shu B-C, Lung F-W, Kuo C-P, Wu W-Y, Yen-Chiao A, Lin Y-J, Lin H-S et al. The pregnancy health and birth outcomes of women who underwent assisted reproductive technology: Results of a national survey. Iranian journal of reproductive medicine 2011;9: 269-276.
16. Lynch A, McDuffie R, Jr., Murphy J, Faber K, Orleans M. Preeclampsia in multiple gestation: the role of assisted reproductive technologies. Obstetrics and gynecology 2002;99: 445-451.
17. Beltran Montoya J, Reyes Munoz E, Cruz Rivera E, Lopez Villasenor B, Francisco de la Jara Diaz J, Herrerias Canedo T. [Adverse perinatal outcomes in Mexican women with twin pregnancy achieved by assisted reproduction techniques vs. spontaneous twin pregnancies]. Ginecologia y obstetricia de Mexico 2012;80: 445-453.
18. Frankenthal D, Hirsh-Yechezkel G, Boyko V, Orvieto R, Ron-El R, Lerner-Geva L, Farhi A. The effect of body mass index (BMI) and gestational weight gain on adverse obstetrical outcomes in pregnancies following assisted reproductive technology as compared to spontaneously conceived pregnancies. Obesity research & clinical practice 2019;13: 150-155.
19. Glavind MT, Forman A, Arendt LH, Nielsen K, Henriksen TB. Endometriosis and pregnancy complications: a Danish cohort study. Fertility and sterility 2017;107: 160-166.
20. Kaveh M, Ghajarzadeh M, Davari Tanha F, Nayeri F, Keramati Z, Shariat M, Ghaheri A. Pregnancy Complications and Neonatal Outcomes in Multiple Pregnancies: A Comparison between Assisted Reproductive Techniques and Spontaneous Conception. International journal of fertility & sterility 2015;8: 367-372.
21. Moguel-Hernandez A, Tietzsch-Escalante P, Iglesias-Leboreiro J, Bernardez-Zapata I, Ramirez-Haua JL, Braverman-Bronstein A. Neonatal complications associated to assisted reproductive techniques at the Hospital Espanol of Mexico. Revista Mexicana de Pediatria 2017;84: 182-188.
22. Kalayci H, Ozdemir H, Alkas D, Cok T, Tarim E. Is primiparity a risk factor for advanced maternal age pregnancies? The journal of maternal-fetal & neonatal medicine : the official journal of the European Association of Perinatal Medicine, the Federation of Asia and Oceania Perinatal Societies, the International Society of Perinatal Obstetricians 2017;30: 1283-1287.
23. Petersen SH, Bergh C, Gissler M, Asvold BO, Romundstad LB, Tiitinen A, Spangmose AL, Pinborg A, Wennerholm U-B, Henningsen A-KA et al. Time trends in placenta-mediated pregnancy complications after assisted reproductive technology in the Nordic countries. American journal of obstetrics and gynecology 2020.
24. Santi E, Nencini G, Cerni A, Greco P, Spelzini F, Tormettino B, Scioscia M. The PLART study: incidence of preterm labor and adverse pregnancy outcomes after assisted reproductive techniques-a retrospective cohort study. Archives of gynecology and obstetrics 2019;300: 911-916.
25. Xu XK, Wang YA, Li Z, Lui K, Sullivan EA. Risk factors associated with preterm birth among singletons following assisted reproductive technology in Australia 2007-2009--a population-based retrospective study. BMC pregnancy and childbirth 2014;14: 406.
26. Vazquez Rodriguez A, Tur Padro R, Martinez San Andres F, Mateo Lopez L, Coroleu Lletget B, Comas Gabriel C, Nolasco Barri Rague P. Influence of age and assisted reproductive techniques in obstetric and perinatal outcomes. Progresos de Obstetricia y Ginecologia 2010;53: 391-398.
27. Yu J, Guo YN, Xie XJ. Pregnancy, birth, and infant outcomes by maternal fertility status: the Massachusetts Outcomes Study of Assisted Reproductive Technology. Medical Journal of Chinese People's Liberation Army 2017;42: 1001-1005.

Not in English, Chinese, French, Spanish, or Portuguese.

1. Aleksanyan A. [COMPLICATIONS OF PREGNANCY, RESULTING FROM ASSISTED REPRODUCTIVE TECHNOLOGY]. Georgian medical news 2017: 63-66.

Overlapped cohort.

1. Luke B, Gopal D, Cabral H, Stern JE, Diop H. Pregnancy, birth, and infant outcomes by maternal fertility status: the Massachusetts Outcomes Study of Assisted Reproductive Technology. American journal of obstetrics and gynecology 2017;217: 327.e321-327.e314.
2. Luke B, Gopal D, Cabral H, Stern JE, Diop H. Adverse pregnancy, birth, and infant outcomes in twins: effects of maternal fertility status and infant gender combinations; the Massachusetts Outcomes Study of Assisted Reproductive Technology. American journal of obstetrics and gynecology 2017;217: 330.e331-330.e315.
3. Luke B, Brown MB, Eisenberg ML, Callan C, Botting BJ, Pacey A, Sutcliffe AG, Baker VL. In Vitro Fertilization and Risk for Hypertensive Disorders of Pregnancy: Associations with Treatment Parameters. American journal of obstetrics and gynecology 2019.
4. Luke B, Brown MB, Nugent C, Gonzalez-Quintero VH, Witter FR, Newman RB. Risk factors for adverse outcomes in spontaneous versus assisted conception twin pregnancies. Fertility and sterility 2004;81: 315-319.
5. Ginstrom Ernstad E, Bergh C, Khatibi A, Kallen KBM, Westlander G, Nilsson S, Wennerholm UB. Neonatal and maternal outcome after blastocyst transfer: A population-based registry study Presented orally at the 36th Society for Maternal-Fetal Medicine Annual Pregnancy Meeting, Atlanta, GA, Feb. 1-6, 2016. American Journal of Obstetrics and Gynecology 2016;214: 378.
6. Stern JE, Liu C-L, Cabral HJ, Richards EG, Coddington CC, Missmer SA, Diop H. Factors associated with increased odds of cesarean delivery in ART pregnancies. Fertility and sterility 2018;110: 429-436.
7. Stern JE, Liu CL, Cabral HJ, Richards EG, Coddington CC, Hwang S, Dukhovny D, Diop H, Missmer SA. Birth outcomes of singleton vaginal deliveries to ART-treated, subfertile, and fertile primiparous women. Journal of Assisted Reproduction and Genetics 2018;35: 1585-1593.
8. Wainstock T, Sheiner E, Yoles I, Sergienko R, Landau D, Harlev A. Fertility treatments and offspring pediatric infectious morbidities: results of a population-based cohort with a median follow-up of 10 years. Fertility and Sterility 2019;112(6):1129-1135.

Wrong outcomes.

1. Haavaldsen C, Tanbo T, Eskild A. Placental weight in singleton pregnancies with and without assisted reproductive technology: a population study of 536,567 pregnancies. Human reproduction (Oxford, England) 2012;27: 576-582.
2. Heo JS, Lee HJ, Lee MH, Choi CW. Comparison of neonatal outcomes of very low birth weight infants by mode of conception: in vitro fertilization versus natural pregnancy. Fertility and Sterility 2019;111: 962-970.
3. Jackson S, Hong C, Wang ET, Alexander C, Gregory KD, Pisarska MD. Pregnancy outcomes in very advanced maternal age pregnancies: The impact of assisted reproductive technology. Fertility and Sterility 2015;103: 76-80.
4. Putterman S, Figueroa R, Garry D, Maulik D. Comparison of obstetric outcomes in twin pregnancies after in vitro fertilization, ovarian stimulation and spontaneous conception. Journal of Maternal-Fetal and Neonatal Medicine 2003;14: 237-240.

Wrong patient population.

1. Li H, Zhu HL, Chang XH, Li Y, Wang Y, Guan J, Cui H. Effects of previous laparoscopic surgical diagnosis of endometriosis on pregnancy outcomes. Chinese Medical Journal 2017;130: 428-433.
2. Cadoret F, Parinaud J, Bettiol C, Pienkowski C, Letur H, Ohl J, Sentilhes L, Papaxanthos A, Winer N, Mathieu d'Argent E et al. Pregnancy outcome in Turner syndrome: A French multi-center study after the 2009 guidelines. European journal of obstetrics, gynecology, and reproductive biology 2018;229: 20-25.
3. De Stefano MG, Rosato E, Marcoccia E, Schiavi MC, Zannini I, Capri O, Galoppi P, Perrone G. Pregnancy in advanced (>= 40) and very advanced (>= 45) reproductive age: Maternal and fetal risks. Giornale Italiano di Ostetricia e Ginecologia 2013;35: 740-746.
4. Fuchs F, Monet B, Ducruet T, Chaillet N, Audibert F. Effect of maternal age on the risk of preterm birth: A large cohort study. Obstetrical and Gynecological Survey 2018;73: 340-342.
5. Canto MJ, Reus A, Cortes S, Ojeda F. Pregnancy outcome in a Spanish population of women beyond age 40 delivered above 32 weeks' gestation. The journal of maternal-fetal & neonatal medicine : the official journal of the European Association of Perinatal Medicine, the Federation of Asia and Oceania Perinatal Societies, the International Society of Perinatal Obstetricians 2012;25: 461-466.
6. Carolan MC, Davey MA, Biro M, Kealy M. Very advanced maternal age and morbidity in Victoria, Australia: A population based study. BMC Pregnancy and Childbirth 2013;13: 80.
7. Chen I, Lalani S, Xie R-H, Shen M, Singh SS, Wen S-W. Association between surgically diagnosed endometriosis and adverse pregnancy outcomes. Fertility and sterility 2018;109: 142-147.
8. Corradetti A, Talebi Chahvar S, Biondini V, Giannubilo SR, Tranquilli AL. PP093 Maternal and fetal outcomes in oocyte donor pregnancies. Pregnancy hypertension 2012;2: 290-291.
9. Toshimitsu M, Nagamatsu T, Nagasaka T, Iwasawa-Kawai Y, Komatsu A, Yamashita T, Osuga Y, Fujii T. Increased risk of pregnancy-induced hypertension and operative delivery after conception induced by in vitro fertilization/intracytoplasmic sperm injection in women aged 40 years and older. Fertility and sterility 2014;102: 1065-1070.e1061.
10. Norrman E, Bergh C, Wennerholm U-B. Pregnancy outcome and long-term follow-up after in vitro fertilization in women with renal transplantation. Human reproduction (Oxford, England) 2015;30: 205-213.
11. Martin V, de Mouzon J. Hypertensive pathologies and egg donation pregnancies: Results of a large comparative cohort study. Fertility and sterility 2016;106: 284-290.
12. Vincent-Rohfritsch A, Le Ray C, Anselem O, Cabrol D, Goffinet F. [Pregnancy in women aged 43 years or older: maternal and perinatal risks]. Journal de gynecologie, obstetrique et biologie de la reproduction 2012;41: 468-475.
13. Harlev A, Walfisch A, Oran E, Har-Vardi I, Friger M, Lunenfeld E, Levitas E. The effect of fertility treatment on adverse perinatal outcomes in women aged at least 40 years. International journal of gynaecology and obstetrics: the official organ of the International Federation of Gynaecology and Obstetrics 2018;140: 98-104.
14. Suzuki S, Miyake H. Obstetric outcomes of elderly primiparous singleton pregnancies conceived by in vitro fertilization compared with those conceived spontaneously. *Reproductive medicine and biology* 2007;6: 219-222.

Wrong study design (case-control studies, reviews, meta-analysis and cross-sectional articles, case reports, editorials, letters to the editor, abstracts, or unpublished studies).

1. Bolijn R, Onland-Moret NC, Asselbergs FW, van der Schouw YT. Reproductive factors in relation to heart failure in women: A systematic review. Maturitas 2017;106: 57-72.
2. Davies MJ, Moore VM, Willson KJ, Van Essen P, Priest K, Scott H, Haan EA, Chan A. Reproductive technologies and the risk of birth defects. Obstetrical and Gynecological Survey 2012;67: 527-528.
3. Finnstrom O, Kallen B, Lindam A, Nilsson E, Nygren K-G, Olausson PO. Maternal and child outcome after in vitro fertilization--a review of 25 years of population-based data from Sweden. Acta obstetricia et gynecologica Scandinavica 2011;90: 494-500.
4. Fuchs F, Monet B, Audibert F, Chaillet N. Impact of advanced maternal age on preterm birth. 2014;210: S382.
5. Hernandez-Diaz S, Werler MM, Mitchell AA. Gestational hypertension in pregnancies supported by infertility treatments: role of infertility, treatments, and multiple gestations. Fertility and Sterility 2007;88: 438-445.
6. Krasniqi MV, Daka Q. Outcomes in IVF conceived pregnancies complicated with hypertensive disorders. Journal of Nepal Paediatric Society 2018;37: 159-163.
7. Lalani S, Choudhry AJ, Firth B, Bacal V, Walker M, Wen SW, Singh S, Amath A, Hodge M, Chen I. Endometriosis and adverse maternal, fetal and neonatal outcomes, a systematic review and meta-analysis. Human reproduction (Oxford, England) 2018;33: 1854-1865.
8. Le Ray C, Pelage L, Seco A, Bouvier-Colle MH, Chantry AA, Deneux-Tharaux C, Epimoms Study G. Risk of severe maternal morbidity associated with in vitro fertilisation: a population-based study. BJOG : an international journal of obstetrics and gynaecology 2019;126: 1033-1041.
9. Lean SC, Derricott H, Jones RL, Heazell AEP. Advanced maternal age and adverse pregnancy outcomes: A systematic review and meta-analysis. PLoS ONE 2017;12: e0186287.
10. Li Y, Zhao S, Yu Y, Ma C, Zheng Y, Niu Y, Wei D, Ma J. Risk factors associated with pre-eclampsia in pregnancies conceived by ART. Reproductive biomedicine online 2019;39: 969-975.
11. Maman E, Lunenfeld E, Levy A, Vardi H, Potashnik G. Obstetric outcome of singleton pregnancies conceived by in vitro fertilization and ovulation induction compared with those conceived spontaneously. Fertility and sterility 1998;70: 240-245.
12. Meints L, Akers D. Effect of conception type on pregnancy outcomes in twin gestations. 2016;23: 271A.
13. Sydsjo G, Lindell Pettersson M, Bladh M, Skoog Svanberg A, Lampic C, Nedstrand E. Evaluation of risk factors' importance on adverse pregnancy and neonatal outcomes in women aged 40 years or older. BMC pregnancy and childbirth 2019;19: 92.
14. Tallo CP, Vohr B, Oh W, Rubin LP, Seifer DB, Haning RV, Jr. Maternal and neonatal morbidity associated with in vitro fertilization. The Journal of pediatrics 1995;127: 794-800.
15. Vuniqi-Krasniqi M, Pacarada M, Daka Q, Dervishi Z, Bimbashi A, Dakaj K. Hypertensive disorders of in-vitro fertilization pregnancies: A study from Kosovo. International journal of reproductive biomedicine (Yazd, Iran) 2018;16: 77-82.
16. Warzecha D, Szymusik I, Grzechocinska B, Cyganek A, Kociszewska-Najman B, Mazanowska N, Madej A, Pazik J, Wielgos M, Pietrzak B. In Vitro Fertilization and Pregnancy Outcomes Among Patients After Kidney Transplantation: Case Series and Single-Center Experience. Transplantation proceedings 2018;50: 1892-1895.
17. Almasi-Hashiani A, Omani-Samani R, Mohammadi M, Amini P, Navid B, Alizadeh A, Khedmati Morasae E, Maroufizadeh S. Assisted reproductive technology and the risk of preeclampsia: an updated systematic review and meta-analysis. BMC pregnancy and childbirth 2019;19: 149.
18. Zullo F, Spagnolo E, Saccone G, Acunzo M, Xodo S, Ceccaroni M, Berghella V. Endometriosis and obstetrics complications: a systematic review and meta-analysis. Fertility and Sterility 2017;108: 667.
19. Luke B, Stern JE, Hornstein MD, Kotelchuck M, Diop H, Cabral H, Declercq ER. Is the wrong question being asked in infertility research? Journal of Assisted Reproduction and Genetics 2016;33: 3-8.
20. Su Y, Zhang Y, Xu Z, Xu X, Xing Q, Zhang L, Xiang HF, Cao YX. Differences between fresh embryo transfer and frozen embryo transfer in asian populations: A meta-analysis. International Journal of Clinical and Experimental Medicine 2018;11: 8820-8830.
21. Berntsen S, Soderstrom-Anttila V, Wennerholm U-B, Laivuori H, Loft A, Oldereid NB, Romundstad LB, Bergh C, Pinborg A. The health of children conceived by ART: 'the chicken or the egg?'. Human reproduction update 2019;25: 137-158.
22. De Weg JMB, De Groot CJ, Pajkrt E, De Boer MA. Recovery of second trimester pre-eclampsia after fetal reduction of a triplet. BMJ Case Reports 2019;12: e227667.
23. Kalra SK, Molinaro TA. The association of in vitro fertilization and perinatal morbidity. Seminars in reproductive medicine 2008;26: 423-435.
24. Maheshwari A, Kalampokas T, Davidson J, Bhattacharya S. Obstetric and perinatal outcomes in singleton pregnancies resulting from the transfer of blastocyst-stage versus cleavage-stage embryos generated through in vitro fertilization treatment: A systematic review and meta-analysis. Fertility and Sterility 2013;100: 1615.
25. Maheshwari A, Pandey S, Raja EA, Shetty A, Hamilton M, Bhattacharya S. Is frozen embryo transfer better for mothers and babies? Can cumulative meta-analysis provide a definitive answer? Human Reproduction Update 2018;24: 35-58.
26. Masoudian P, Nasr A, de Nanassy J, Fung-Kee-Fung K, Bainbridge SA, El Demellawy D. Oocyte donation pregnancies and the risk of preeclampsia or gestational hypertension: a systematic review and metaanalysis. American journal of obstetrics and gynecology 2016;214: 328-339.
27. Masouridou S, Mamopoulos A, Mavromatidis G, Karagiannis V. Endometriosis and perinatal outcome - A systematic review of the literature. Current Women's Health Reviews 2012;8: 121-130.
28. Qin J, Wang H, Sheng X, Liang D, Tan H, Xia J. Pregnancy-related complications and adverse pregnancy outcomes in multiple pregnancies resulting from assisted reproductive technology: a meta-analysis of cohort studies. Fertility and sterility 2015;103: 1492-1497.
29. Qin JZ, Pang LH, Li MJ, Fan XJ, Huang RD, Chen HY. Obstetric complications in women with polycystic ovary syndrome: A systematic review and meta-analysis. Reproductive Biology and Endocrinology 2013;11: 56.
30. Roque M, Valle M, Sampaio M, Geber S. Obstetric outcomes after fresh versus frozen-thawed embryo transfers: A systematic review and meta-analysis. JBRA assisted reproduction 2018;22: 253-260.
31. Sha T, Wang X, Cheng W, Yan Y. A meta-analysis of pregnancy-related outcomes and complications in women with polycystic ovary syndrome undergoing IVF. Reproductive biomedicine online 2019;39: 281-293.
32. Tandulwadkar SR, Lodha PA, Mangeshikar NT. Obstetric complications in women with IVF conceived pregnancies and polycystic ovarian syndrome. Journal of Human Reproductive Sciences 2014;7: 13-18.
33. Jackson RA, Gibson KA, Wu YW, Croughan MS. Perinatal outcomes in singletons following in vitro fertilization: a meta-analysis. Obstetrics and gynecology 2004;103: 551-563.
34. Allen VM, Wilson RD, Cheung A. Pregnancy outcomes after assisted reproductive technology. Journal of obstetrics and gynaecology Canada : JOGC = Journal d'obstetrique et gynecologie du Canada : JOGC 2006;28: 220-250.
35. Bartsch E, Medcalf KE, Park AL, Ray JG, Al-Rubaie ZTA, Askie LM, Berger H, Blake J, Graves L, Kingdom JC et al. Clinical risk factors for pre-eclampsia determined in early pregnancy: Systematic review and meta-analysis of large cohort studies. The BMJ 2016;353: i1753.
36. Bohlmann MK, Fritzsching B, Luedders DW, Hornemann A, Gopel W, Poschl J, Diedrich K, Griesinger G. [Impact of assisted reproduction on obstetrics and neonatology]. Zeitschrift fur Geburtshilfe und Neonatologie 2009;213: 221-227.
37. Chen X-K, Wen SW, Bottomley J, Smith GN, Leader A, Walker MC. In vitro fertilization is associated with an increased risk for preeclampsia. Hypertension in pregnancy 2009;28: 1-12.
38. Gelbaya TA. Short and long-term risks to women who conceive through in vitro fertilization. Human fertility (Cambridge, England) 2010;13: 19-27.
39. Grewe S, Grewe C, Diedrich K. Obstetric risks after assisted reproduction. Gynakologe 2019;52: 659-666.
40. Luke B. Pregnancy and birth outcomes in couples with infertility with and without assisted reproductive technology: with an emphasis on US population-based studies. American journal of obstetrics and gynecology 2017;217: 270-281.
41. Okun N, Sierra S, Genetics C, Special C. Pregnancy outcomes after assisted human reproduction. Journal of obstetrics and gynaecology Canada : JOGC = Journal d'obstetrique et gynecologie du Canada : JOGC 2014;36: 64-83.
42. Omani-Samani R, Alizadeh A, Almasi-Hashiani A, Mohammadi M, Saman M, Navid B, Khedmati Morasae E, Amini P. Risk of preeclampsia following assisted reproductive technology: systematic review and meta-analysis of 72 cohort studies. The journal of maternal-fetal & neonatal medicine : the official journal of the European Association of Perinatal Medicine, the Federation of Asia and Oceania Perinatal Societies, the International Society of Perinatal Obstetricians 2018: 1-331.
43. Perez-Lopez FR, Calvo-Latorre J, Alonso-Ventura V, Bueno-Notivol J, Martinez-Dominguez SJ, Chedraui P, Health Outcomes SAP. Systematic review and meta-analysis regarding the association of endometriosis and preeclampsia in women conceiving spontaneously or through assisted reproductive technology. Pregnancy hypertension 2018;14: 213-221.
44. Pandey S, Shetty A, Hamilton M, Bhattacharya S, Maheshwari A. Obstetric and perinatal outcomes in singleton pregnancies resulting from IVF/ICSI: a systematic review and meta-analysis. Human reproduction update 2012;18: 485-503.
45. Qin J, Liu X, Sheng X, Wang H, Gao S. Assisted reproductive technology and the risk of pregnancy-related complications and adverse pregnancy outcomes in singleton pregnancies: A meta-analysis of cohort studies. Fertility and Sterility 2016;105: 73-85e76.
46. Savasi VM, Mandia L, Laoreti A, Cetin I. Maternal and fetal outcomes in oocyte donation pregnancies. Human Reproduction Update 2016;22: 620-633.
47. Storgaard M, Loft A, Bergh C, Wennerholm UB, Soderstrom-Anttila V, Romundstad LB, Aittomaki K, Oldereid N, Forman J, Pinborg A. Obstetric and neonatal complications in pregnancies conceived after oocyte donation: a systematic review and meta-analysis. BJOG : an international journal of obstetrics and gynaecology 2017;124: 561-572.
48. Thomopoulos C, Salamalekis G, Kintis K, Andrianopoulou I, Michalopoulou H, Skalis G, Archontakis S, Argyri O, Tsioufis C, Makris TK et al. Risk of hypertensive disorders in pregnancy following assisted reproductive technology: overview and meta-analysis. Journal of clinical hypertension (Greenwich, Conn) 2017;19: 173-183.
49. Thomopoulos C, Tsioufis C, Michalopoulou H, Makris T, Papademetriou V, Stefanadis C. Assisted reproductive technology and pregnancy-related hypertensive complications: a systematic review. Journal of human hypertension 2013;27: 148-157.
50. Wennerholm U-B, Bergh C. Perinatal outcome in children born after assisted reproductive technologies. Upsala journal of medical sciences 2020: 1-9.
51. Barros Delgadillo JC, Alvarado Mendez LM, Gorbea Chavez V, Villalobos Acosta S, Sanchez Solis V, Gavino Gavino F. [Perinatal results in pregnancies obtained with embryo transfer in vitro fertilization: a case-control study]. Ginecologia y obstetricia de Mexico 2006;74: 626-639.
52. Chaveeva P, Carbone IF, Syngelaki A, Akolekar R, Nicolaides KH. Contribution of method of conception on pregnancy outcome after the 11-13 weeks scan. Fetal diagnosis and therapy 2011;30: 9-22.
53. Diedrich K, Banz-Jansen C, Ludwig AK. Risks of pregnancy and outcome in children born after ART. Journal fur Reproduktionsmedizin und Endokrinologie 2011;8: 108-111.
54. Giannakou K, Evangelou E, Papatheodorou SI. Genetic and non-genetic risk factors for pre-eclampsia: umbrella review of systematic reviews and meta-analyses of observational studies. Ultrasound in obstetrics & gynecology : the official journal of the International Society of Ultrasound in Obstetrics and Gynecology 2018;51: 720-730.
55. Lehnen H, Schafer S, Reineke T, Puchooa A, Maiwald R, Zechner U. Twin pregnancies conceived spontaneously and by ART (Assisted Reproductive Technologies) a retrospective analysis and review. Geburtshilfe und Frauenheilkunde 2011;71: 669-676.
