# Supplementary material for: Assisted reproductive technology and hypertensive disorders of pregnancy: systematic review and meta-analyses
Source: BMC Pregnancy Childbirth. 2021 Jun 28;21:449. doi: 10.1186/s12884-021-03938-8 (PMC8240295; doi:10.1186/s12884-021-03938-8)

Additional file 5. Forest plots comparing preeclampsia in IVF/ICSI pregnancies and spontaneous pregnancies and funnel plots for publication bias in meta-analyses with 10 or more studies.

Figure 1. Forest plot comparing preeclampsia in a) IVF/ICSI singleton pregnancies and b) ICSI singleton pregnancies in comparison to spontaneous pregnancies.


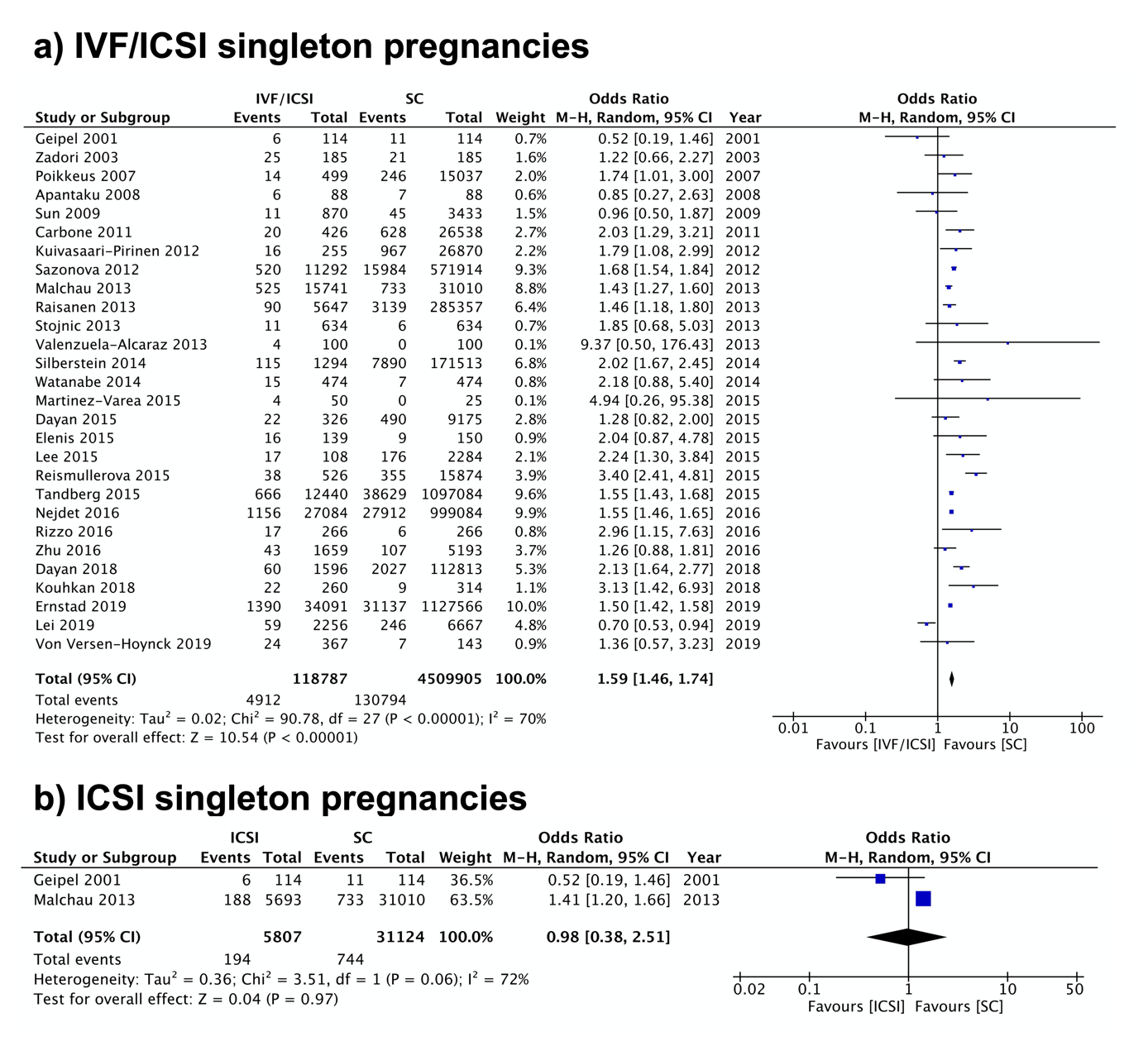


Figure 2. Forest plot comparing preeclampsia in IVF/ICSI multiple pregnancies and spontaneous pregnancies.


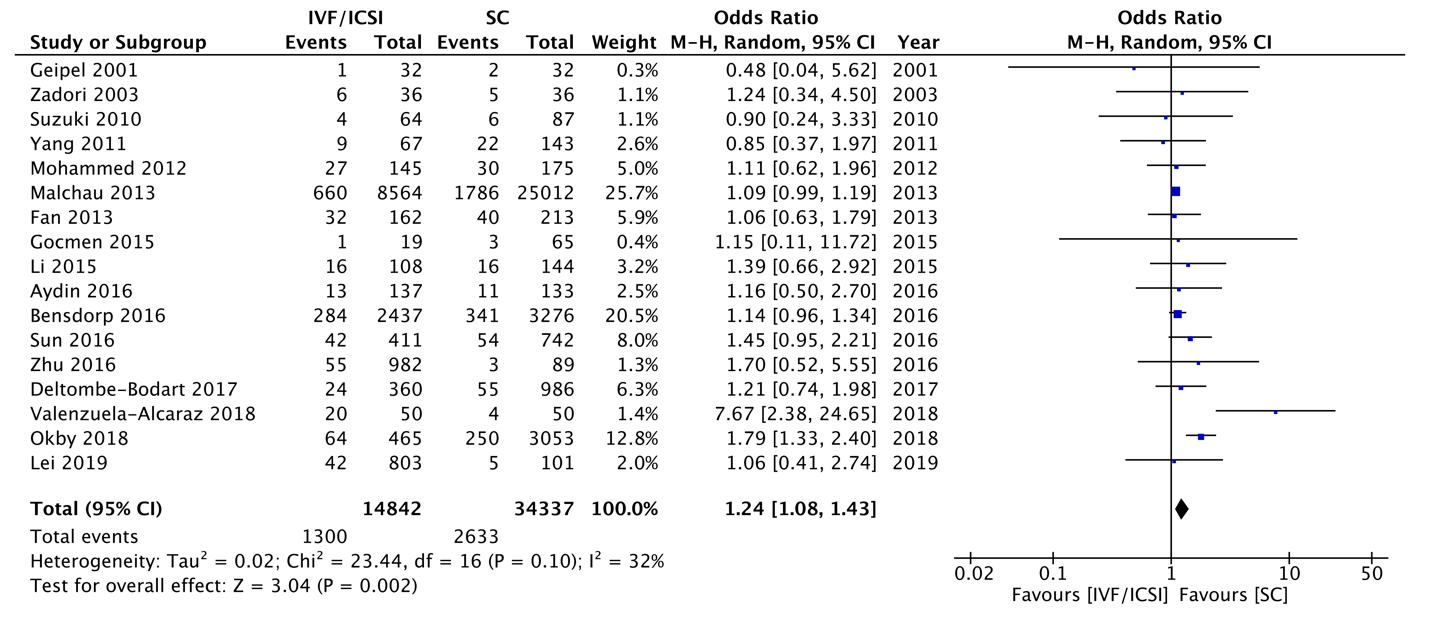


Figure 3. Forest plot comparing preeclampsia in a) IVF multiple pregnancies and B) ICSI multiple pregnancies in comparison to spontaneous pregnancies.


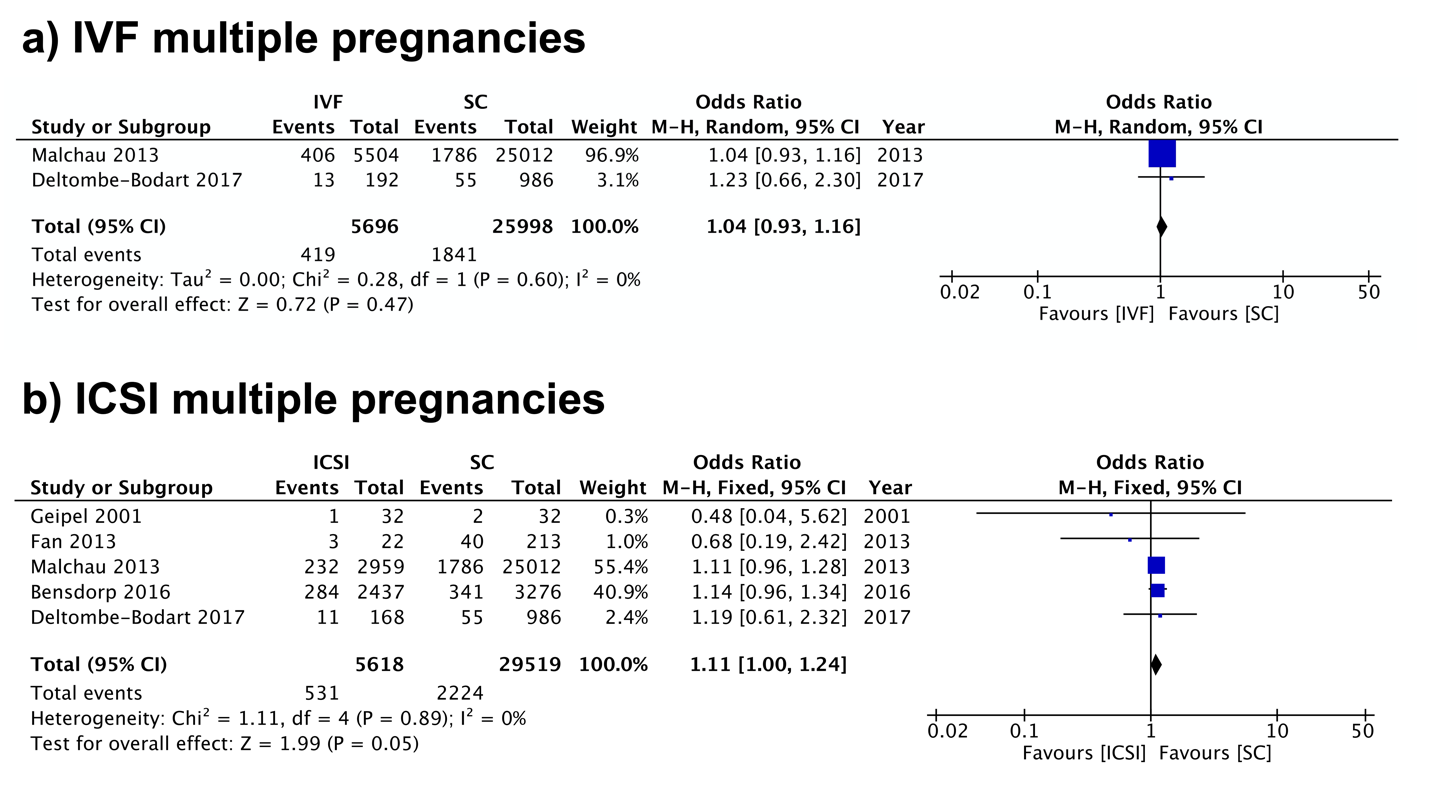


Figure 4. Forest plot comparing preeclampsia in singleton pregnancies resulting from a) fresh embryo transfer and b) frozen embryo transfer in comparison to spontaneous pregnancies.


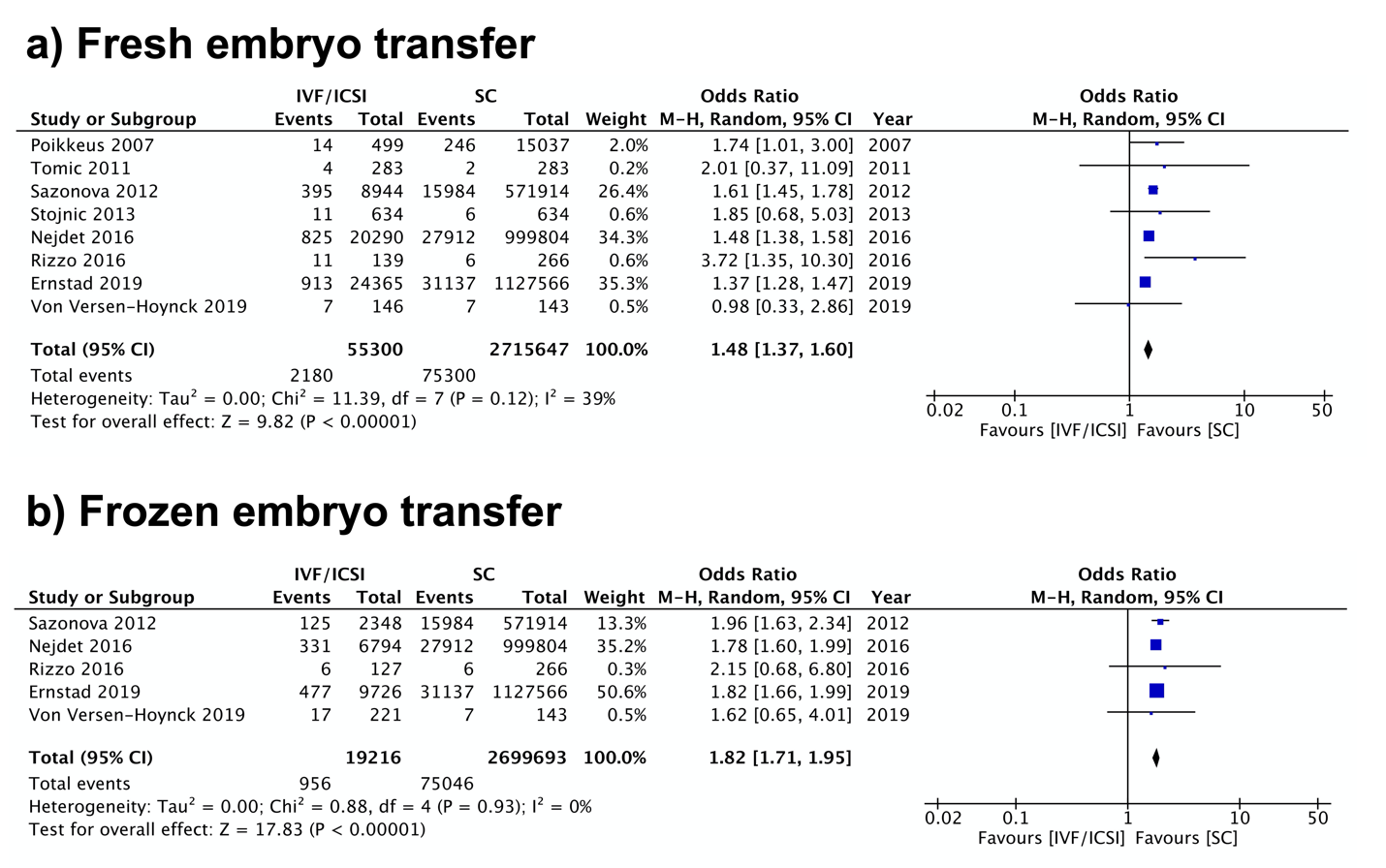


Figure 5. Forest plot comparing preeclampsia in singleton pregnancies resulting from oocyte donation and spontaneous pregnancies.


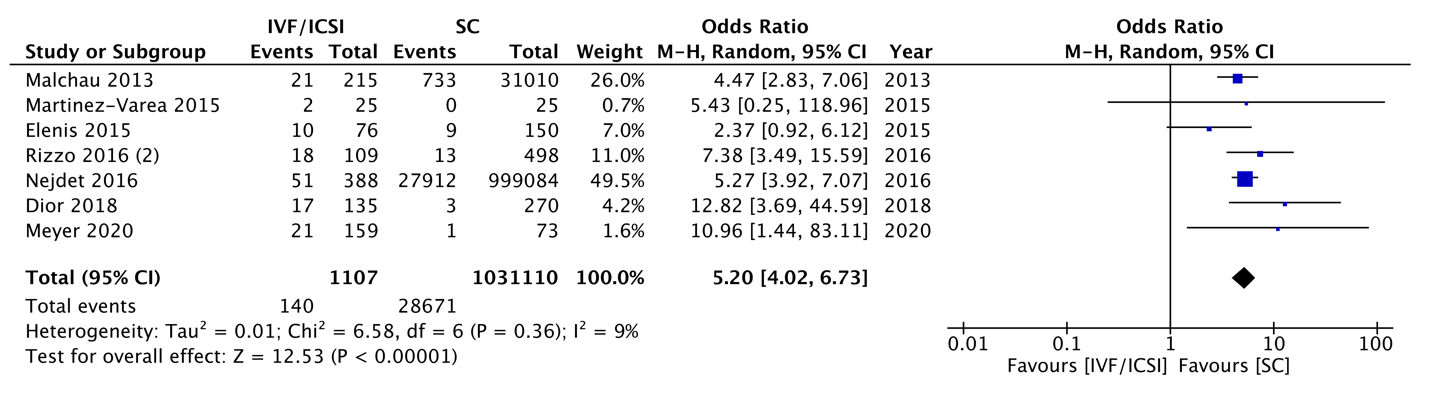


Figure 6. Assessing Publication Bias Using Funnel Plots – IVF/ICSI Singleton Pregnancies: Hypertensive Disorders of Pregnancy.


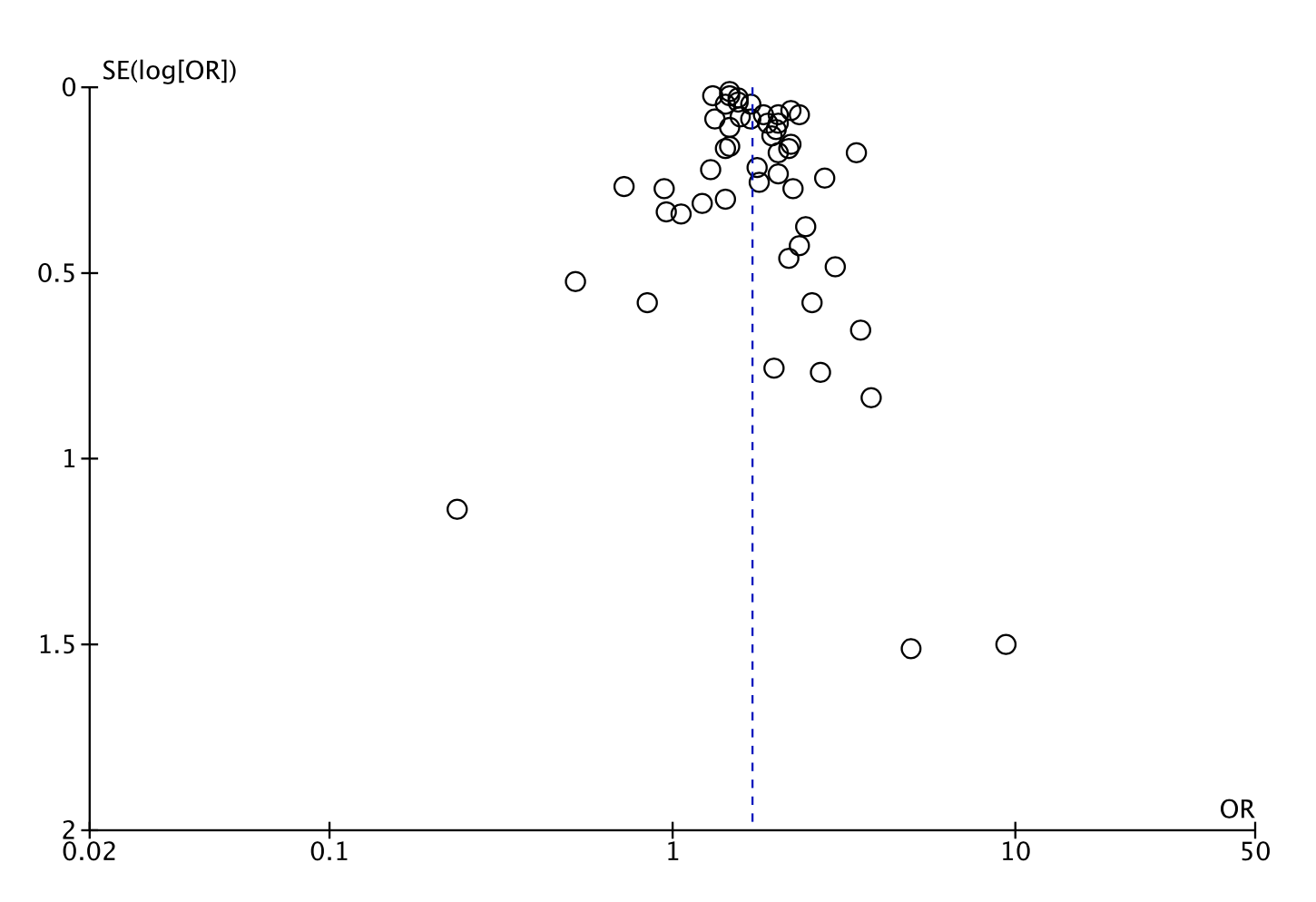


Figure 7. Assessing Publication Bias Using Funnel Plots – IVF/ICSI Multiple Pregnancies: Hypertensive Disorders of Pregnancy.


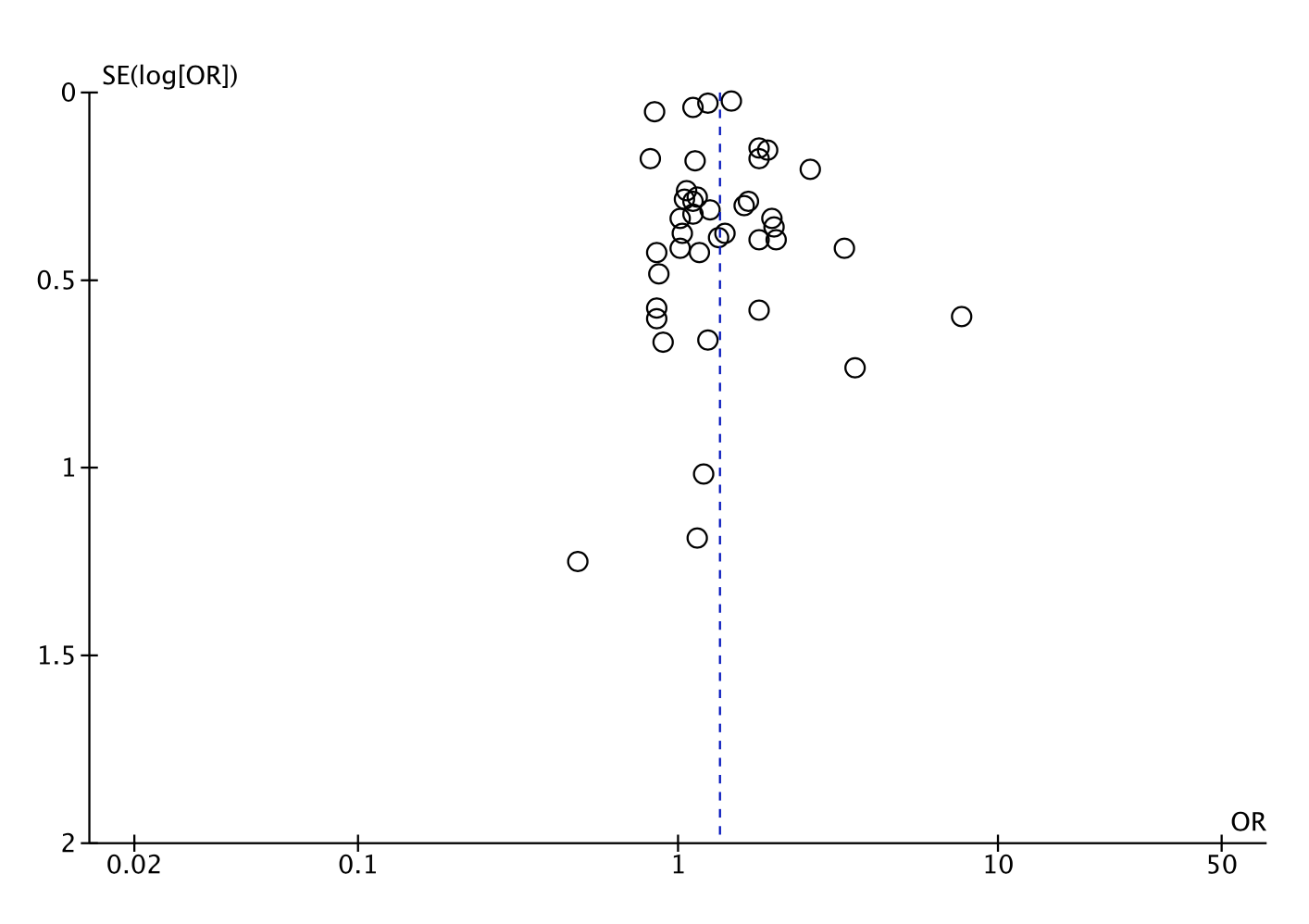


Figure 8. Assessing Publication Bias Using Funnel Plots – ICSI Multiple Pregnancies: Hypertensive Disorders of Pregnancy.


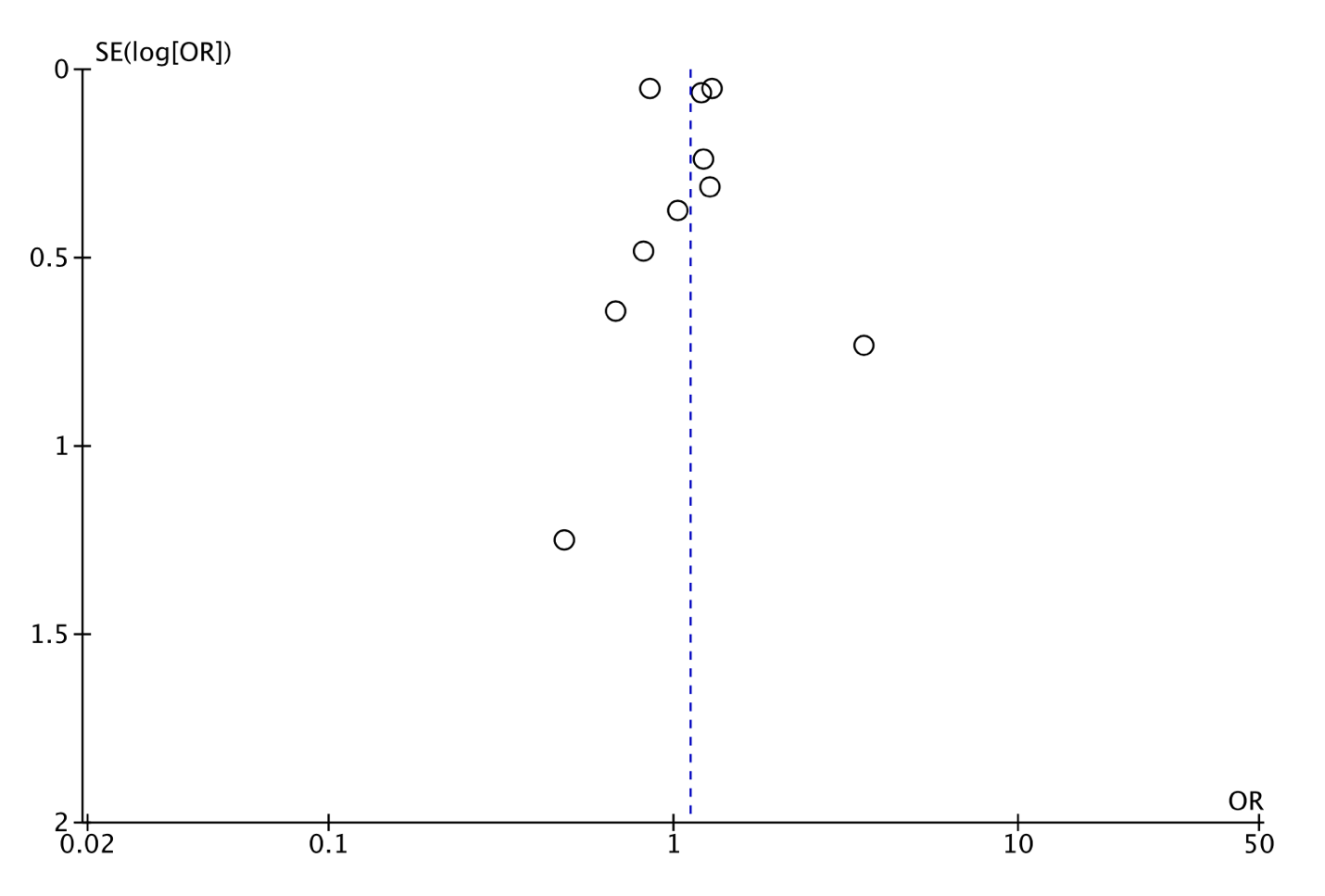


Figure 9. Assessing Publication Bias Using Funnel Plots – Fresh Embryo Transfer: Hypertensive Disorders of Pregnancy.


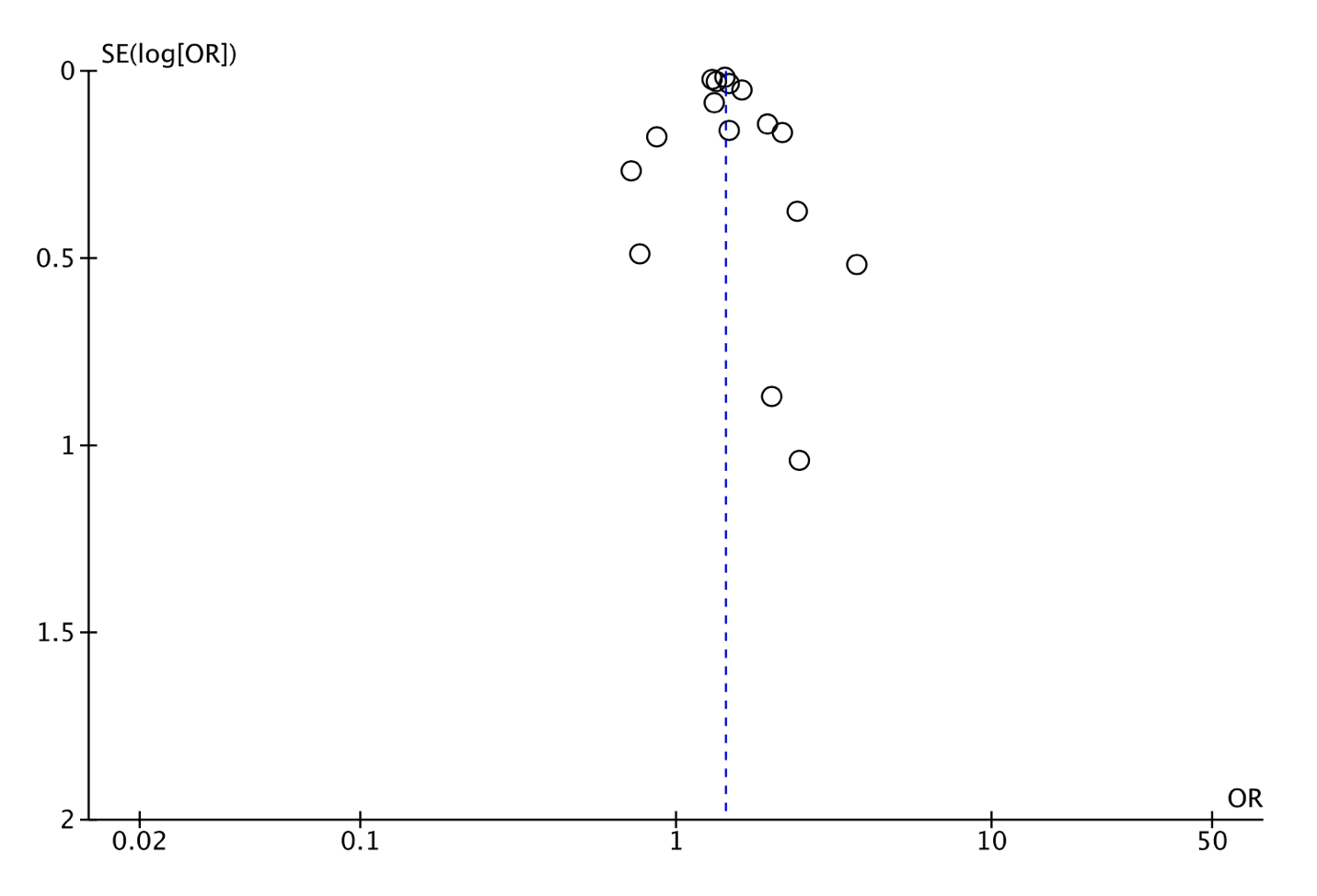


Figure 10. Assessing Publication Bias Using Funnel Plots – IVF/ICSI Singleton Pregnancies: Preeclampsia.


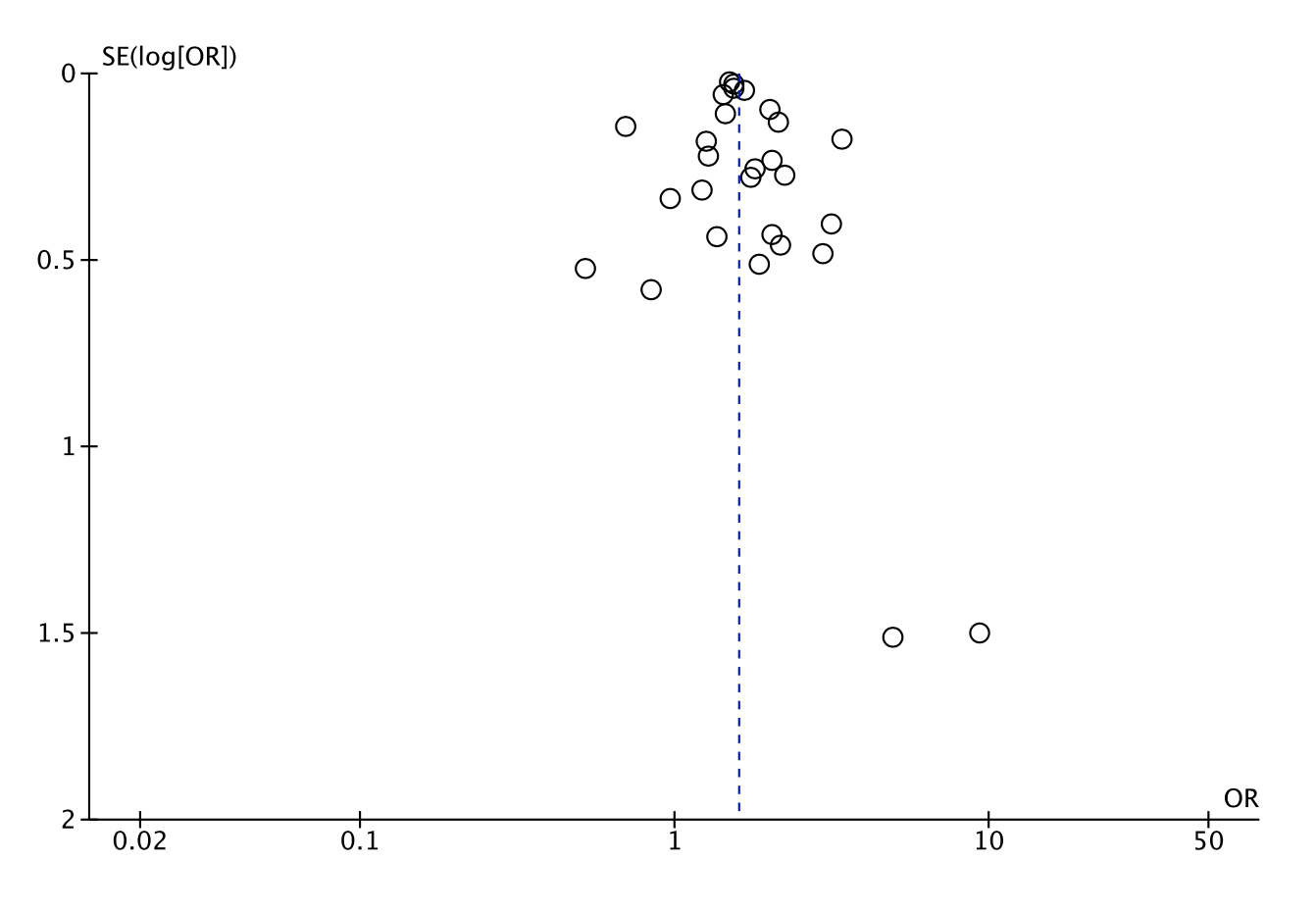


Figure 11. Assessing Publication Bias Using Funnel Plots – IVF/ICSI Multiple Pregnancies: Preeclampsia.


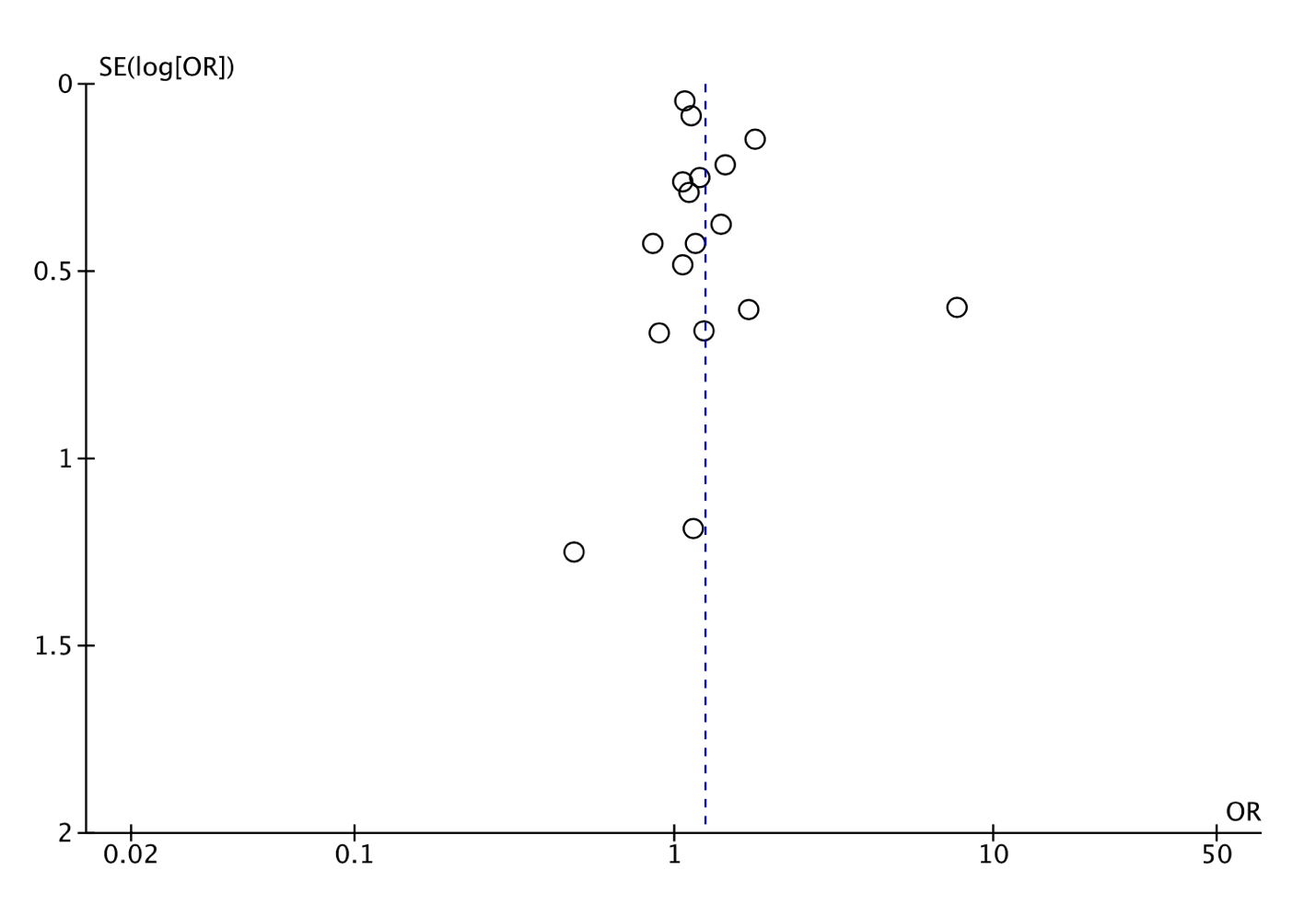

Supplement: Supplementary file 5 — Additional file 5. Forest plots for preeclampsia and funnel plots. Forest plots comparing preeclampsia in IVF/ICSI pregnancies and spontaneous pregnancies and funnel plots for publication bias in meta-analyses with 10 or more studies. [file 12884_2021_3938_MOESM5_ESM.docx]
