# Supplementary material for: Assisted reproductive technology and hypertensive disorders of pregnancy: systematic review and meta-analyses
Source: BMC Pregnancy Childbirth. 2021 Jun 28;21:449. doi: 10.1186/s12884-021-03938-8 (PMC8240295; doi:10.1186/s12884-021-03938-8)
Supplement: Supplementary file 6 — Additional file 6. Sensitivity analysis. A list of highest and lowest overall odds ratios after removing individual studies. [file 12884_2021_3938_MOESM6_ESM.docx]

Additional file 6. Sensitivity analysis.

| Experimental | OR; 95% CI | Lowest OR after excluding one study; 95% CI | Highest OR after excluding one study; 95% CI |
| --- | --- | --- | --- |
|  | Hypertensive Disorders of Pregnancy | | |
| IVF/ICSI singleton | 1.70 (1.60 - 1.80) | 1.67 (1.58 - 1.77) | 1.72 (1.62 - 1.82) |
| IVF singleton | 1.55 (1.23 - 1.94) | 1.28 (1.17 - 1.40) | 1.70 (1.16 - 2.49) |
| ICSI singleton | 1.52 (1.28 - 1.80) | 1.37 (1.27 - 1.49) | 1.57 (1.27 - 1.93) |
| IVF/ICSI multiple | 1.34 (1.20 - 1.50) | 1.31 (1.17 - 1.46) | 1.38 (1.25 - 1.52) |
| IVF multiple | 1.13 (0.98 - 1.29) | 1.04 (0.94 - 1.14) | 1.22 (1.13 - 1.31) |
| ICSI multiple | 1.11 (0.91 - 1.36) | 1.06 (0.85 - 1.34) | 1.25 (1.16 - 1.35) |
| Fresh embryo transfer singleton | 1.43 (1.33 - 1.53) | 1.40 (1.31 - 1.51) | 1.45 (1.34 - 1.57) |
| Frozen embryo transfer singleton | 1.74 (1.58 - 1.92) | 1.70 (1.54 - 1.89) | 1.81 (1.63 - 2.00) |
| Oocyte donation singleton | 4.42 (3.00 - 6.51) | 4.12 (2.77 - 6.13) | 5.01 (4.12 - 6.10) |
| Oocyte donation multiple | 2.62 (2.46 - 2.79) | 2.62 (2.45 - 2.79) | 3.07 (1.94 - 4.86) |
|  | Preeclampsia | | |
| IVF/ICSI singleton | 1.59 (1.46 - 1.74) | 1.54 (1.43 - 1.67) | 1.64 (1.53 - 1.77) |
| ICSI singleton | 0.98 (0.38 - 2.51) | 0.52 (0.19 - 1.46) | 1.41 (1.20 - 1.66) |
| IVF/ICSI multiple | 1.24 (1.08 - 1.43) | 1.12 (1.04 - 1.21) | 1.30 (1.10 - 1.54) |
| IVF multiple | 1.04 (0.93 - 1.16) | 1.04 (0.93 - 1.16) | 1.23 (0.66 - 2.30) |
| ICSI multiple | 1.11 (1.00 - 1.24) | 1.10 (0.96 - 1.26) | 1.12 (1.01 - 1.24) |
| Fresh embryo transfer singleton | 1.48 (1.37 - 1.60) | 1.43 (1.34 - 1.53) | 1.52 (1.44 - 1.61) |
| Frozen embryo transfer singleton | 1.82 (1.71 - 1.95) | 1.80 (1.68 - 1.93) | 1.84 (1.70 - 2.00) |
| Oocyte donation singleton | 5.20 (4.02 - 6.73) | 4.97 (3.75 - 6.60) | 5.57 (3.85 - 8.06) |
